# Transcriptional transitions in *Nicotiana benthamiana* leaves upon induction of oil synthesis by WRINKLED1 homologs from diverse species and tissues

**DOI:** 10.1186/s12870-015-0579-1

**Published:** 2015-08-08

**Authors:** Åsa Grimberg, Anders S. Carlsson, Salla Marttila, Rishikesh Bhalerao, Per Hofvander

**Affiliations:** Department of Plant Breeding, Swedish University of Agricultural Sciences, Växtskyddsvägen 1, P.O. Box 101, SE-23053 Alnarp, Sweden; Department of Plant Protection Biology, Swedish University of Agricultural Sciences, Alnarp, Sweden; Department of Forest Genetics and Plant Physiology, Umeå Plant Science Centre, Umeå, Sweden

## Abstract

**Background:**

Carbon accumulation and remobilization are essential mechanisms in plants to ensure energy transfer between plant tissues with different functions or metabolic needs and to support new generations. Knowledge about the regulation of carbon allocation into oil (triacylglycerol) in plant storage tissue can be of great economic and environmental importance for developing new high-yielding oil crops. Here, the effect on global gene expression as well as on physiological changes in leaves transiently expressing five homologs of the transcription factor *WRINKLED1* (*WRI1*) originating from diverse species and tissues; *Arabidopsis thaliana* and potato (*Solanum tuberosum*) seed embryo, poplar (*Populus trichocarpa*) stem cambium, oat (*Avena sativa*) grain endosperm, and nutsedge (*Cyperus esculentus*) tuber parenchyma, were studied by agroinfiltration in *Nicotiana benthamiana*.

**Results:**

All *WRI1* homologs induced oil accumulation when expressed in leaf tissue. Transcriptome sequencing revealed that all homologs induced the same general patterns with a drastic shift in gene expression profiles of leaves from that of a typical source tissue to a source-limited sink-like tissue: Transcripts encoding enzymes for plastid uptake and metabolism of phosphoenolpyruvate, fatty acid and oil biosynthesis were up-regulated, as were also transcripts encoding starch degradation. Transcripts encoding enzymes in photosynthesis and starch synthesis were instead down-regulated. Moreover, transcripts representing fatty acid degradation were up-regulated indicating that fatty acids might be degraded to feed the increased need to channel carbons into fatty acid synthesis creating a futile cycle. RT-qPCR analysis of leaves expressing Arabidopsis *WRI1* showed the temporal trends of transcripts selected as ‘markers’ for key metabolic pathways one to five days after agroinfiltration. Chlorophyll fluorescence measurements of leaves expressing Arabidopsis *WRI1* showed a significant decrease in photosynthesis, even though effect on starch content could not be observed.

**Conclusions:**

This data gives for the first time a general view on the transcriptional transitions in leaf tissue upon induction of oil synthesis by WRI1. This yields important information about what effects WRI1 may exert on global gene expression during seed and embryo development. The results suggest why high oil content in leaf tissue cannot be achieved by solely transcriptional activation by WRI1, which can be essential knowledge in the development of new high-yielding oil crops.

**Electronic supplementary material:**

The online version of this article (doi:10.1186/s12870-015-0579-1) contains supplementary material, which is available to authorized users.

## Background

Among different forms of carbon storage, triacylglycerol (TAG; oil) is the most energy dense, yielding more than double amount of energy on a per weight basis as compared to starch. Due to the increased global need for plant oil derived products for food but also for non-food applications to decrease our dependence on fossil oil, the interest in mechanisms for regulation of carbon allocation into oil in plant storage tissues has gained a lot of attention and can be of great economic and environmental importance in the process of developing new high-yielding oil crops [1, 2]. To be available for TAG synthesis, carbons from sucrose must first be converted to pyruvate through cytosolic or plastidic glycolytic pathways and at some point be imported into the plastid to yield acetyl-coenzyme A (CoA) which feeds fatty acid (FA) synthesis with carbon backbones. FAs are subsequently transported out to the endoplasmic reticulum where TAG assembly takes place [3].

The seed-specific, loss-of-function mutant *wrinkled1* of *Arabidopsis thaliana* with 80 % reduction in oil content and increased levels of sucrose but not of starch in mature seeds was by enzymatic analyses shown to be impaired in the allocation of carbon into glycolysis [4]. The corresponding gene, *AtWRINKLED1* (*AtWRI1,* At3g54320), encodes a protein with two APETALA2-ethylene responsive element-binding protein (AP2/EREBP) motifs that is a signature for plant-specific transcription factors involved in a wide range of developmental processes [5, 6]. Gene expression analyses using microarrays have further revealed that some of the down-regulated genes in seeds of the *wri1* mutant are involved in glycolysis and FA synthesis, rather than in TAG assembly [7]. Moreover, AtWRI1 has been shown to bind to the AW-box sequence in upstream regions of several genes involved in FA synthesis and glycolysis [8–10].

Homologs to *AtWRI1* have been identified in oil-dense embryonic tissues of several other plant species as rape seed (*Brassica napus)* and maize (*Zea mays*) [11, 12]. A homolog to *AtWRI1* was also associated with the large difference in carbon allocation to oil between oil palm (*Elaeis guineensis*) and date palm (*Phoenix dactylifera*) mesocarps with the former mainly storing carbon as oil, the latter as sugar [13]. Today’s global plant oil production is mainly dependent on TAG storage capacity in oil palm fruits and the embryos of soybean, sunflower, and oilseed rape [14]. Studies trying to reveal the regulatory mechanisms behind oil accumulation have mainly focused on embryonic tissues of seeds [11, 12, 15], even though fruit mesocarp and seed endosperm tissues have also been studied [13, 16, 17]. However, the potential to produce oil in already high-yielding but not usually oil dense tissues like cereal grain endosperm and underground tuber or root parenchyma, by directing carbon allocation from starch or sugar into oil by genetic engineering, deserves further attention due to the important applied benefits.

Among plant species producing underground tubers, only one is known to have genotypes with high amount of oil (up to 30 % of dry weight), *i.e.* yellow nutsedge [18, 19]. Among the cereals, oat is the only species known to store relatively high amount of oil (up to 18 % of grain weight) in the grain endosperm [20, 21]. In both these tissues, homologs to the *AtWRI1* were expressed during tuber parenchyma and grain endosperm development, respectively (unpublished data, and [22]). Another interesting tissue for potential oil production is from trees due to the vast amount of wood processed in the pulp industry from which FAs are already an important byproduct [23]. Studies on perennial plants have indicated that lipids can accumulate transiently in the cambium upon transition to dormancy [24, 25] and a homolog to the *AtWRI1* has been identified in poplar from available genomic resources [26]. Therefore, the role of *WRI1*-like genes for directing carbon flow into oil synthesis and accumulation could be extended to include diverse types of plant tissues.

In oilseed species like Arabidopsis, oil accumulation is part of the embryo development and seed maturation process which is governed by master regulators such as LEC1, LEC2, FUS3 and ABI3 [15, 27]. WRI1 is linked to this upstream regulatory network and has been shown to be a downstream target and specify the action of LEC2 towards FA synthesis [10, 27]. However, homologs to these upstream regulators were not found in the mesocarp of oil palm, which suggests that the regulatory networks in non-embryo tissues are different to those in developing seed embryos [13]. In this study, we investigated the effect on global gene expression in leaves where oil accumulation was induced by expression of *WRI1* homologs originating from diverse species and tissues (Arabidopsis and potato seed embryo, poplar stem cambium, oat grain endosperm, and nutsedge tuber parenchyma) using a transient gene expression system with agroinfiltration of *Nicotiana benthamiana*. A time study of oil and starch content as well as RT-qPCR and photosynthesis measurements in leaves expressing Arabidopsis *WRI1* between one to five days after infiltration was done to further characterize and complement the transcriptional transitions observed at five days after infiltration.

## Results and discussion

### All homologs were *WRI1*-like

**Fig. 1:**
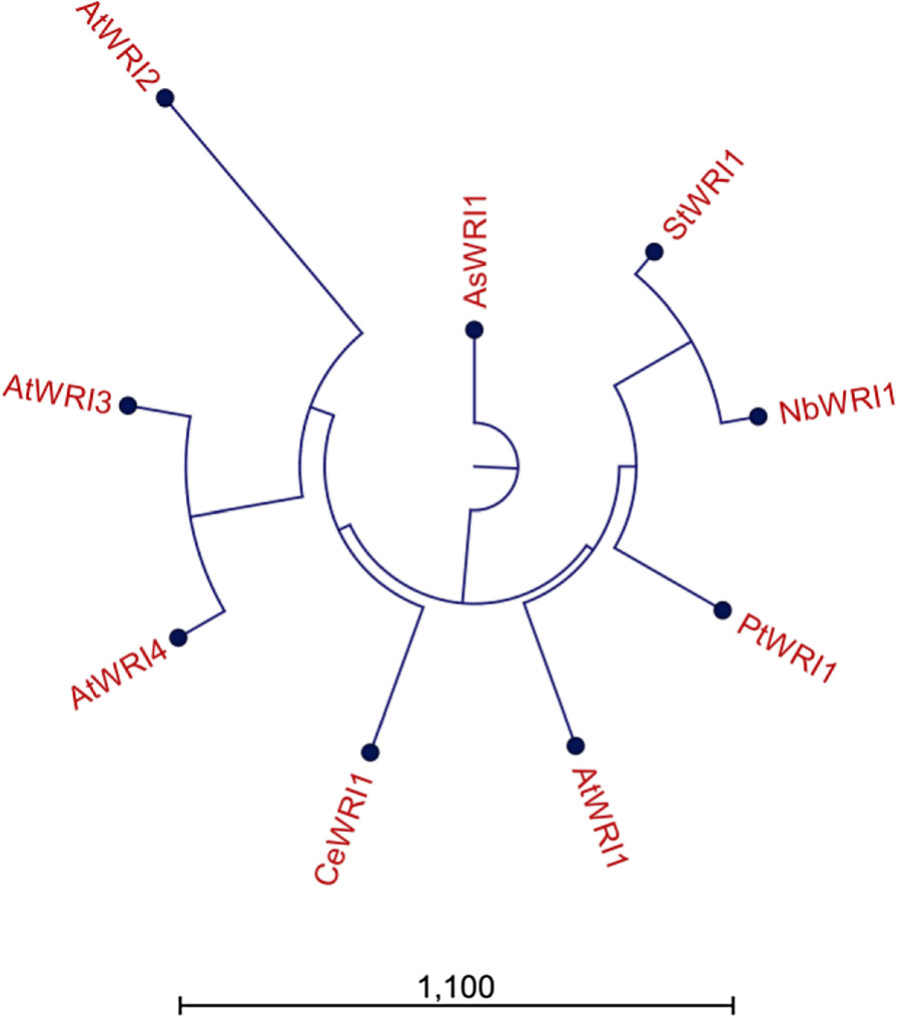
Phylogeny tree of *WRI1* homologs. Phylogeny analysis based on full-length cDNA sequences of *WRI* homologs from Arabidopsis (*AtWRI1*, *2*, *3*, and *4*), potato embryo (*StWRI1em*), tobacco (*NbWRI1*), poplar stem (*PtWRI1ca*), oat endosperm (*AsWRI1es*), and nutsedge tuber parenchyma (*CeWRI1tp*)

The *WRI1* gene from Arabidopsis and four additional homologs from different species representing three dicotyledons and two monocotyledons were used in the present study (for complete cDNA sequences, see Additional file 1). These selected homologs are expressed in different types of tissues that all have induction of oil accumulation at certain developmental or temporal stages. The Arabidopsis gene is expressed in the embryo during seed development as is the potato homolog, and the poplar homolog is expressed in the cambium of the tree. The oat homolog is expressed in grain endosperm and the nutsedge homolog in underground stem tuber. From a phylogenetic tree based on homology of full-length cDNA it was clear that the novel *WRI1* homologs of this study were more similar to Arabidopsis *WRI1* than to either Arabidopsis *WRI2, WRI3,* or *WRI4* [28] which are more similar to each other than to any of the other presented homologs (Fig. 1). It was also evident from this phylogeny analysis that the two homologs of *WRI1* originating from monocot species were more similar to each other than to the dicot *WRI1* homologs. The WRI1 orthologs and homologs so far studied have a high degree of identity and similarity on an amino acid level in the region spanning the two AP2 domains but with a high degree of divergence in the N-and C-terminus [29] which was also true for the WRI1 homologs of this study (Additional file 2). However, outside the DNA binding domains there was a high divergence among the different homologs although it was evident that species which are more closely related have a higher degree of amino acid homology and identity. This in particular applies to the derived amino acid sequences of the *N. benthamiana* and *S. tuberosum* homologs of WRI1 with 82 % identity in contrast to the homolog from *C. esculentus* which had only 45 % identity to that of *N. benthamiana*. The different homologs are hereafter named *AtWRI1*, *StWRI1em*, *PtWRI1ca*, *AsWRI1es*, and *CeWRI1tp* abbreviated after the species and tissue of origin (embryo, cambium, endosperm, and tuber parenchyma, respectively).

### All WRI1-like homologs induced oil accumulation in leaves

**Fig. 2:**
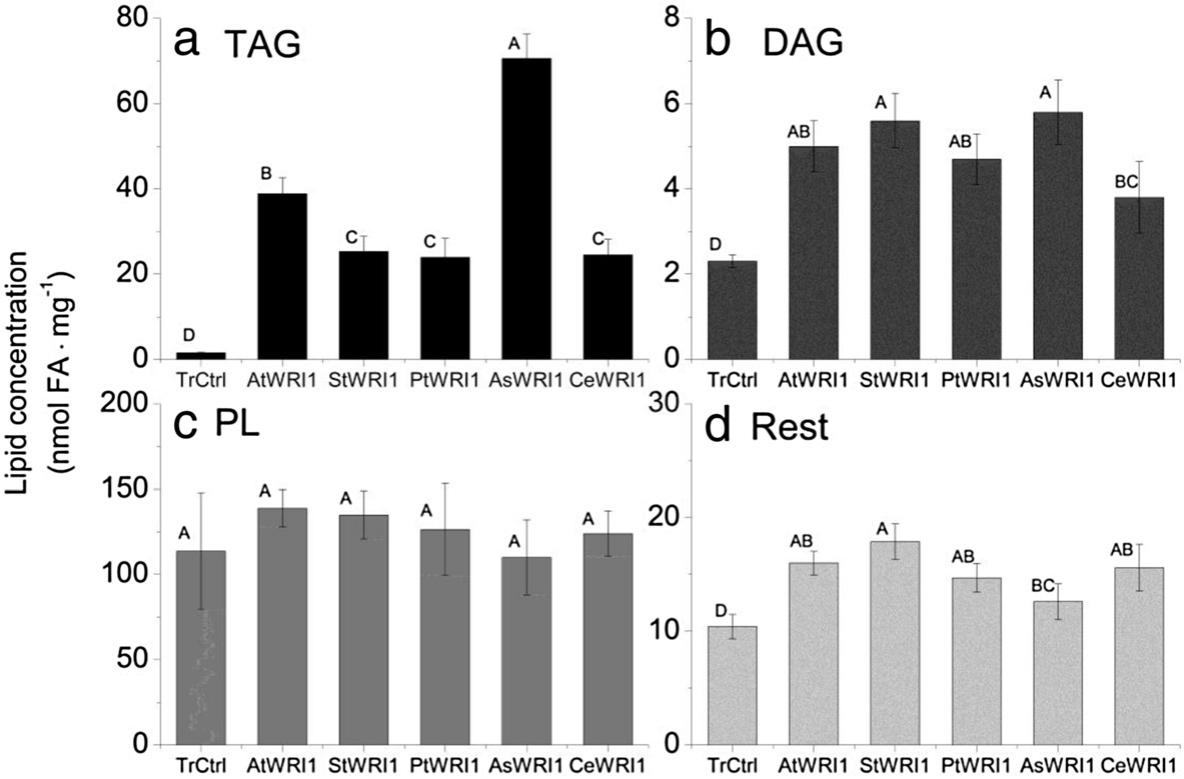
Leaf lipid concentrations. Concentration of different lipid classes (TAG; triacylglycerol (**a**), DAG; diacylglycerol (**b**), PL; polar lipids (**c**), and other lipids (**d**)) given as nmol fatty acids (FA) on dry weight basis of *N. benthamiana* leaves expressing *WRI1* from Arabidopsis embryo (*AtWRI1*), potato embryo (*StWRI1em*), oat endosperm (*AsWRI1es*), poplar stem (*PtWRI1ca*), nutsedge tuber parenchyma (*CeWRI1tp*), and transformed control (TrCtrl) five days after infiltration. Results are the mean from three biological replicates ± standard deviation. Letters distinguish significant different means according to Tukey’s test at level *P* ≤ 0.05

All WRI1 homologs in this study were shown to drastically induce oil (triacylglycerol; TAG) accumulation when transiently expressed in leaves of *N. benthamiana* by agroinfiltration (Fig. 2a, Additional file 3). Leaves expressing the *AsWRI1es* showed the highest oil concentration (2.2 % of leaf dw as compared to 0.05 % in transformed control) followed by *AtWRI1, StWRI1em*, *PtWRI1ca*, *and CeWRI1tp* (Fig. 2a). This result shows that the function of WRI1 is highly conserved across species and storage tissues even though not of typical oil-dense embryo type. Also diacylglycerol levels increased in leaves upon expression of the *WRI1* homologs (Fig. 2b). Polar lipids (which are important constituents of membranes) were the most abundant in leaves but did not show any difference between leaves expressing the different *WRI1* homologs or to transformed control (Fig. 2c). The leaves expressing the *WRI1* homologs appeared stressed with chlorosis, in particular for leaves expressing *StWRI1em*. Dry matter contents of leaves were not significantly different from transformed control (12 %), except for leaves expressing the *AsWRI1es* with higher dry matter (16 %, see Additional file 4).

**Fig. 3:**
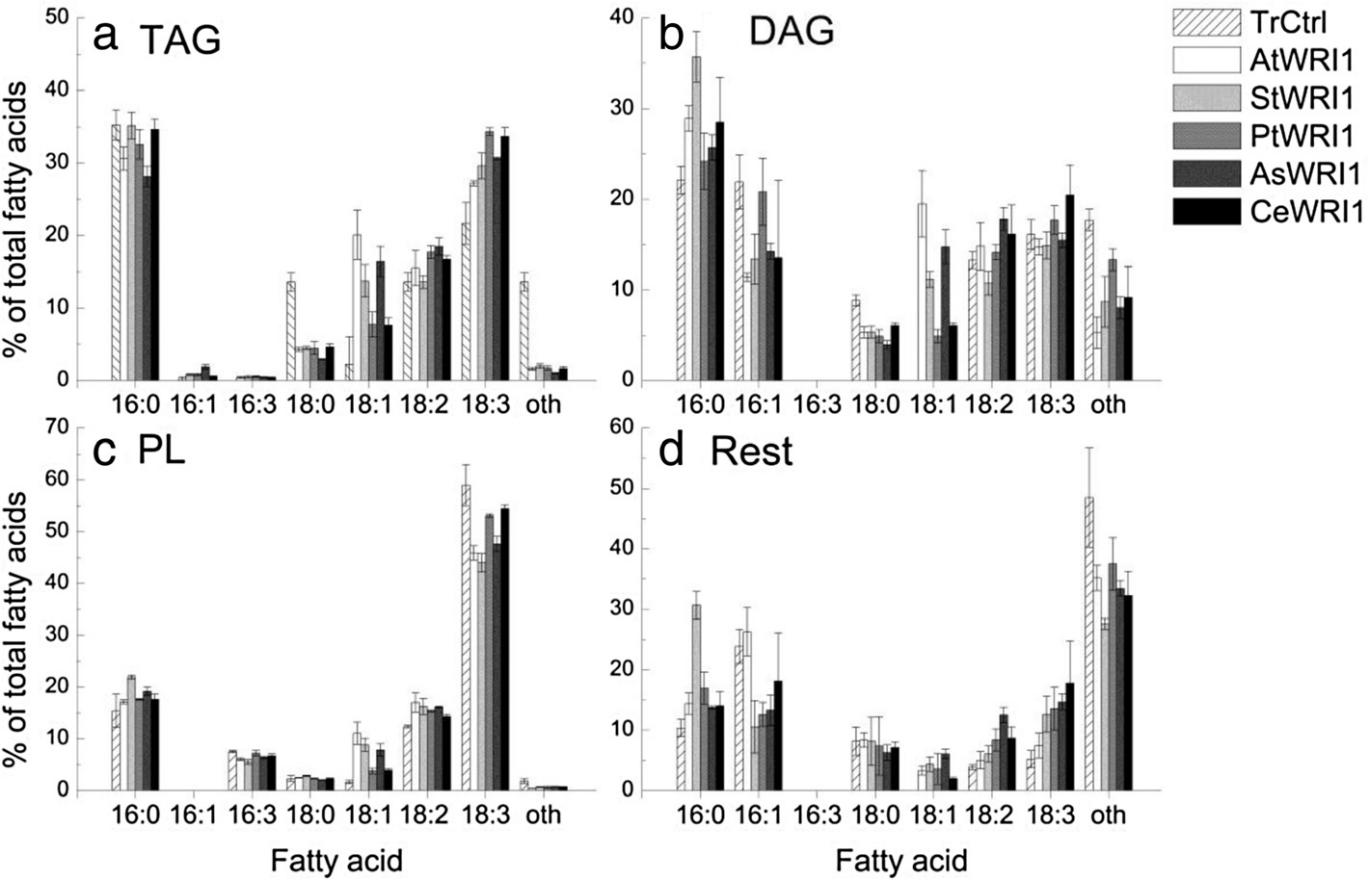
Fatty acid profiles of lipids. TAG; triacylglycerol (**a**), DAG; diacylglycerol (**b**), PL; polar lipids (**c**), and other lipids (**d**) in *N. benthamiana* leaves expressing *WRI1* from Arabidopsis embryo (*AtWRI1*), potato embryo (*StWRI1em*), oat endosperm (*AsWRI1es*), poplar stem (*PtWRI1ca*), nutsedge tuber parenchyma (*CeWRI1tp*), and transformed control (TrCtrl) five days after infiltration. Results are the mean from three biological replicates ± standard deviation

The FA composition of TAG changed upon expression of *WRI1* homologs with lower proportions of 18:0 and in general higher proportions of 18:1, as compared to transformed control (Fig. 3a). In the polar lipid fraction of leaves, the largest observed differences in FA profile upon expression of *WRI1* homologs were in general increased proportions of 18:1 and decreased 18:3 (Fig. 3c).

**Fig. 4:**
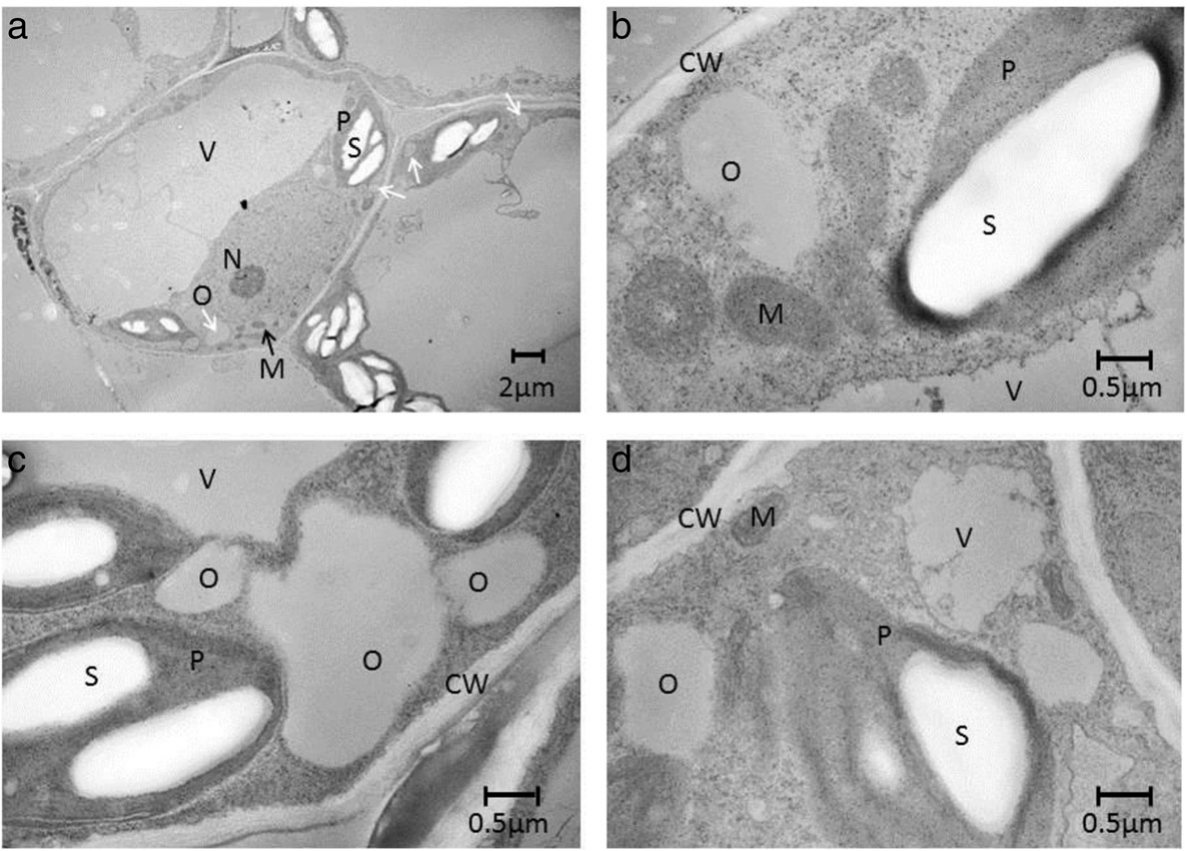
Transmission electron microscopy. Photos of *N. benthamiana* leaves expressing *WRI1* from oat endosperm (*AsWRI1es*; **a**, **b**) and Arabidopsis embryo (*AtWRI1*, **c**). Transformed control leaf (**d**) five days after infiltration. CW; cell wall, P; plastid, S; starch, O; oil (white arrows), M; mitochondria, V; vacuole, N; nucleus

Since oil accumulation was highest in leaves expressing *AsWRI1es* and *AtWRI1*, the localization and structural properties of oil were only analyzed for those (Fig. 4a-c) together with transformed control leaves (Fig. 4d). TEM analysis indicated that the accumulated oil was localized to the cytosol and was most probably present as oil droplets and not as distinct organized oil-bodies surrounded by oil-body bound proteins. This is consistent with that no up-regulation of transcripts encoding oil-body proteins was observed in the transcriptome data (see below). However, differences in oil quantity could not be clearly visualized by this method. Expression of oleosins in Arabidopsis leaves already expressing phospholipid:diacylglycerol acyltransferase promoted oil accumulation giving 6.4 % oil of leaf dw and also changed the structural storage form into organized oil-bodies [30], and combining oleosins with WRI1 and diacylglyceroltransferase1 in *N. tabacum* leaves gave even higher oil content, 15 % by leaf dw [31]. This demonstrates the potential of combining a transcription factor with genes involved in both TAG synthesis and protection against mobilization, for achieving high oil content in leaves. For clarification, the aim of our study was not to achieve the highest oil content possible but instead to map the transcriptional changes induced by WRI1 alone in leaf tissue.

### Transcriptome sequencing data

Leaves expressing the different *WRI1* homologs and transformed control tissue were subjected to transcriptome sequencing five days after agro-infiltration, which is generally used as a suitable time for sufficient transient gene expression and impact on metabolism [32–35]. Upon expression of *WRI1* homologs in the *N. benthamiana* leaf tissue, notable differences in the number of differentially expressed genes as compared to transformed control were observed, but all five homologs induced a similar pattern in core metabolic pathways. Expression of *StWRI1em* in leaves induced the highest number of differentially expressed genes for both up-regulated and down-regulated transcripts, followed by *AtWRI1*, *PtWRI1ca*, *AsWRI1es,* and *CeWRI1tp* (Additional file 5). However, since the expression levels of the different *WRI1* homologs varied significantly in leaves (RPKM values of *CeWRI1tp*; 29382, *AsWRI1es*; 25132, *AtWRI1;* 7419, *StWRI1em;* 2712, and *PtWRI1ca;* 1597), no conclusions regarding species-specific differential gene expression induced by those different homologs could be made from this study. Fold-changes of gene expression given here are therefore the averages from leaves expressing the different WRI1 homologs. Interesting to note though, is that the expression levels of the different *WRI1* homologs did not correlate either to oil content or number of differential expressed genes in leaves. In fact, the number of differentially expressed genes was instead correlated with phylogenetic similarity of the different *WRI1* homologs to the *N. benthamiana WRI1*, suggesting that the most closely related *StWRI1em* yielded the highest and *CeWRI1tp* the lowest number of differentially expressed genes in leaves.

The lists of differentially expressed genes in leaves (determined according to chosen criteria, see Methods) upon expression of the different *WRI1* homologs had in general a similar overall pattern for core metabolic pathways (differentially expressed genes are found in Additional file 6). Here, these general trends are described where the functional equivalence of each gene was assigned according to the closest homolog of Arabidopsis (At-numbers of all genes discussed can be found in Additional file 6). The effect on global gene expression in leaves induced by WRI1 homologs was in general strong with many genes shown to be highly differentially regulated. Some genes were virtually switched ‘on’, going from almost non-detectable in transformed control tissue to a relatively high expression in *WRI1*-infiltrated tissue. This illustrates the strong effect of using a constitutively expressed transcription factor to achieve a drastic change of the gene expression program of a certain tissue. The results also reveal notable similarities in the effect on transcriptional regulation exerted by WRI1 homologs coming from such diverse plant species and tissues as were included in this study. It is important to note that it is not possible to distinguish whether the observed effects on gene expression in leaves five days after agroinfiltration were due to direct or indirect effects of WRI1 expression. Furthermore, this study only discusses induced changes at the transcript level and cannot state whether these changes is also correlated to protein levels.

#### Carbon directed to the plastid

**Fig. 5:**
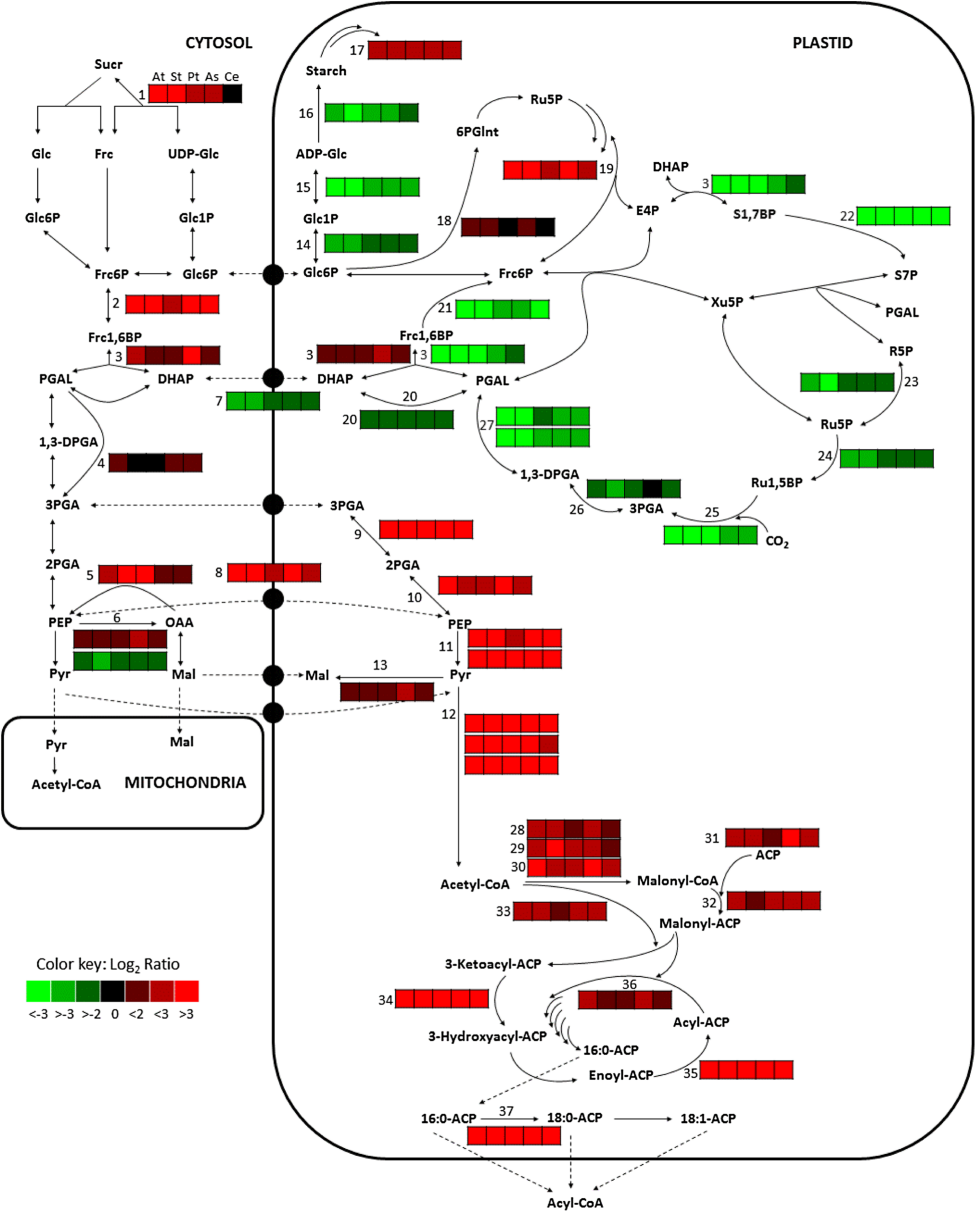
Heat map of differential gene expression involved in central carbon metabolism. Values given are log_2_ of the ratios between RPKM of leaves expressing *WRI1* and transformed control five days after infiltration. The order of leaf samples expressing the five different *WRI1* homologs are given for the box numbered 1: *AtWRI1*, *StWRI1em*, *PtWRI1ca*, *AsWRI1es*, and *CeWRI1tp*. Abbreviations: E4P; erythrose 4-phosphate, Sucr; sucrose, Glc; glucose, Frc; fructose, Frc6P; fructose 6-phosphate, Glc6P; glucose 6-phosphate, Frc1,6BP; fructose 1,6-bisphosphate, UDP; uridine diphosphate, Glnt; gluconate, R5P; ribose 5-phosphate, Ru5P; ribulose 5-phosphate, Ru1,5BP; ribulose 1,5-bisphosphate, PGAL; phosphoglyceraldehyde, PGA; phosphoglycerate, DPGA; diphosphoglycerate, PEP; phosphoenolpyruvate, Pyr; pyruvate, OAA, oxaloacetate, Mal; malate, ADP; adenosinediphospho-, S7P; sedoheptulose 7-phosphate, S1,7BP; sedoheptulose 1,7- bisphosphate, Xu5P; xylulose 5-phosphate. Enzymes: 1. Sucrose synthase, 2. PPi-dependent fructose-6-phosphate 1-phosphotransferase, 3. Fructose-bisphosphate aldolase, 4. NADP-dependent glyceraldehyde-3-phosphate dehydrogenase, 5. Phosphoenolpyruvate carboxykinase, 6. Phosphoenolpyruvate carboxylase, 7. Triosephosphate/phosphate translocator, 8. Phosphoenolpyruvate/phosphate translocator, 9. Phosphoglycerate mutase, 10. Enolase, 11. Pyruvate kinase (alpha and beta subunit), 12. Pyruvate dehydrogenase (subunit E1, E2, E3), 13. Malic enzyme, 14. Phosphoglucomutase, 15. ADP-glucose pyrophosphorylase large subunit, 16. Starch synthase 4, 17. β-amylase, 18. Glucose-6-phosphate dehydrogenase, 19. Transaldolase, 20. Triose-phosphate isomerase, 21. Fructose-1,6-bisphosphatase, 22. Sedoheptulose 1,7-bisphosphatase, 23. Ribose-5-phosphate isomerase, 24. Phosphoribulokinase, 25. RubisCO, 26. Phosphoglycerate kinase, 27. Glyceraldehyde-3-phosphate dehydrogenase (subunit A and B), 28. Carboxyl transferase α (CT) subunit of acetyl-CoA carboxylase (ACC), 29. Biotin carboxylase (BC) of ACC, 30. Biotin carboxyl carrier protein 2 (BCCP) of ACC, 31. Acyl carrier protein (ACP), 32. Malonyl-CoA:ACP malonyltransferase, 33. 3-ketoacyl-ACP synthase III (KAS III), 34. 3-ketoacyl-ACP reductase, 35. Enoyl-ACP reductase, 36. 3-ketoacyl-ACP synthase I (KAS I), 37. 3-ketoacyl-ACP synthase II (KAS II)

The oil-inducing effect of WRI1 in Arabidopsis seeds has been attributed to its enhancing expression of genes encoding enzymes in glycolysis as well as FA synthesis, rather than in TAG assembly [4, 7, 10, 36]. Many of the genes, as for biotin carboxylase carrier protein, KASI, enoyl-ACP reductase, ACP, fatty acid desaturase 2, plastid pyruvate kinase, and plastid pyruvate dehydrogenase E1α, which are down-regulated in the Arabidopsis mutant *wri1* as compared to in wild-type seeds, are within the set of genes involved in FA synthesis and glycolysis shown to have a bell-shaped expression pattern during wild-type seed development [7]. On the contrary, genes involved in TAG assembly are not differentially expressed in the *wri1* seeds and do not belong to the ‘bell-shaped group’ of genes in the wild-type. Studies on Arabidopsis seeds also showed that the activities of several glycolytic enzymes are much decreased in the *wri1* as compared to wild-type seeds [4, 37]. Therefore, it was not surprising that leaves expressing *WRI1* homologs in our study indeed had a much higher expression of genes involved in glycolysis and FA synthesis pathways, as compared to in transformed control (Fig. 5, Additional file 6). *WRI1* expression also induced a shift at the transcript level in the organelle distribution of glycolysis with carbon flux being directed to the plastid. Genes encoding enzymes of the upper part of cytosolic glycolysis; sucrose synthase (except for in leaves expressing *CeWRI1tp*), PPi-dependent fructose-6-phosphate (Frc-6P) 1-phosphotransferase, and Frc-bisphosphate aldolase, and the lower part of plastidic glycolysis; phosphoglycerate mutase, enolase, the α and β subunit of pyruvate kinase, and all three subunits (E1-E3) of pyruvate dehydrogenase were highly up-regulated (average fold-changes between 4 and 46, highest for plastidic phosphoglycerate mutase) in leaves expressing *WRI1*. Up-regulated were also transcripts of the plastid translocator for phosphoenolpyruvate (9-fold), thereby probably ensuring cytosolic glycolysis up to phosphoenolpyruvate followed by uptake into the plastid for further plastidic glycolysis. Interestingly, this transcript pattern of organelle shift for glycolysis is very similar to that of Arabidopsis seeds during development [7], indicating that this might be a signature of a plant tissue in transformation from a photosynthetic source to heterotrophic oil accumulation. This pattern is also in agreement with the observed decreased flux of carbon through the lower part of plastidic glycolysis from phosphoenolpyruvate and downwards in developing embryos of the *wri1* Arabidopsis mutant as determined by ^13^C metabolic flux analyses [36].

#### Increased storage synthesis

Pyruvate from glycolysis is converted by pyruvate dehydrogenase into acetyl-CoA which is the two-carbon precursor for *de novo* FA synthesis in the plastid. Many genes encoding enzymes involved in FA synthesis were shown to be up-regulated in leaves expressing the *WRI1* homologs (Fig. 5, Additional file 6) again well in agreement with other studies (see above). Transcripts encoding all subunits of acetyl-CoA carboxylase (carboxyl transferase, biotin carboxylase, and biotin carboxylase carrier protein), acyl carrier protein (ACP), malonyl-CoA:ACP malonyltransferase and 3-ketoacyl-ACP synthase III (KASIII), 3-ketoacyl-ACP synthase I (KAS I) were all up-regulated (4 to 7-fold) in leaves expressing *WRI1* homologs. A very high up-regulation was observed for the reductases of 3-ketoacyl-ACP and enoyl-ACP of the fatty acid synthase complex (15-fold). One transcript that was up-regulated 278-fold in leaves expressing the *WRI1* homologs, with very low expression in the transformed control tissue, was clearly annotated as a KAS but phylogenetic analyses (based on both DNA and amino acid sequences) did not allow for an obvious designation to KASI or II in Arabidopsis. However, no other KAS genes exist in *N. benthamiana* and therefore this transcript most probably encodes the enzyme activity corresponding to that of KASII in Arabidopsis. The high up-regulation of this transcript indicates the importance of the elongation of 16:0-ACP to 18:0-ACP for increased TAG synthesis.

Genes encoding enzyme activities in TAG assembly were previously not usually associated with WRI1 regulation (see above). However, a few transcripts related to TAG assembly were in this study found to be up-regulated in leaves upon *WRI1* expression (Additional file 6). One transcript annotated to represent phosphatidylcholine diacylglycerol cholinephosphotransferase (PDCT) was up-regulated (5-fold) in leaves expressing the *WRI1* homologs. PDCT is involved in the regulation of TAG composition by catalyzing the inter-conversion of diacylglycerol and phosphatidylcholine [38], and interestingly the encoding gene was previously shown to be down-regulated in the triple mutant of Arabidopsis with reduced expression of *WRI1*, *WRI3*, and *WRI4* [28]. Transcripts encoding the enzymes phospholipase C and D were also up-regulated (average fold changes of 27 and 2, respectively). Both types of lipases are (as also for PDCT) thought to be important for the conversion of phosphatidylcholine to diacylglycerol [3]. Therefore, PDCT and/or phospholipases might therefore be the enzymes defining the observed shift in FA composition of TAG upon induction of oil accumulation. Furthermore, a transcript encoding glycerol-3P dehydrogenase, catalyzing the synthesis of glycerol-3P (the TAG backbone) from dihydroxyacetonephosphate was up-regulated 8-fold. One plastidic isoform of glycerol-3P dehydrogenase in Arabidopsis (At2g40690) was previously shown to be important for the metabolism of plastid-localized glycerolipids [39] and is probably not associated with TAG synthesis (which takes place outside the plastid). However, the glycerol-3P dehydrogenase shown to be up-regulated in our study (corresponding to At5g40610) was encoding another plastidic isoform [40] which might be important for TAG backbone supply since it is associated with gene expression during seed development in Arabidopis (Additional file 7), and glycerol-3P can pass the plastid envelope through a permease which was recently shown to play an important role for lipid content of Arabidopsis embryos [41]. Interestingly, the gene encoding the plastidic glycerol-3P dehydrogenase found to be up-regulated in our study was also identified to be one of 18 putative target genes of maize (*Zea mays*) WRI1 [42].

Many genes involved in amino acid metabolism (synthesis, transport and protein processing) were differentially expressed upon expression of *WRI1* homologs in our study (Additional file 6) which could possibly indicate increased activities involved with protein storage accumulation. Transcripts encoding vacuolar sorting receptor was 2-fold up-regulated while that of vacuolar processing enzyme as much as 1400-fold up-regulated. Other examples of up-regulated transcripts encoded membrane amino acid transporters (40-fold) and endoplasmic reticulum to Golgi vesicle mediated transport of amino acids (7-fold). Transcripts encoding asparagine synthetase and glutamate synthase was up-regulated (85 and 4-fold, respectively), while on the other hand that of chloroplastic glutamine synthetase was down-regulated (4-fold). Transcripts annotated as amino transferases of aspartate and alanine were up-regulated (2-fold). However, it was difficult to completely dissect the meaning of these differentially expressed genes as pathways were up-regulated or down-regulated during seed development in Arabidopsis [43], which could fit with *WRI1* expression although changes in amino acid metabolism and nitrogen remobilization is also consistent with other patterns such as stress or senescence. Changes in expression of genes in amino acid metabolism are also indicative as markers for changes in general metabolism in the leaf tissue [44]. It is interesting to note that increased levels of certain amino acids were actually observed in maize kernels overexpressing the *ZmWRI1a* [42] which can indicate an association of WRI1 with storage protein synthesis.

#### Decreased photosynthesis and starch formation

**Fig. 6:**
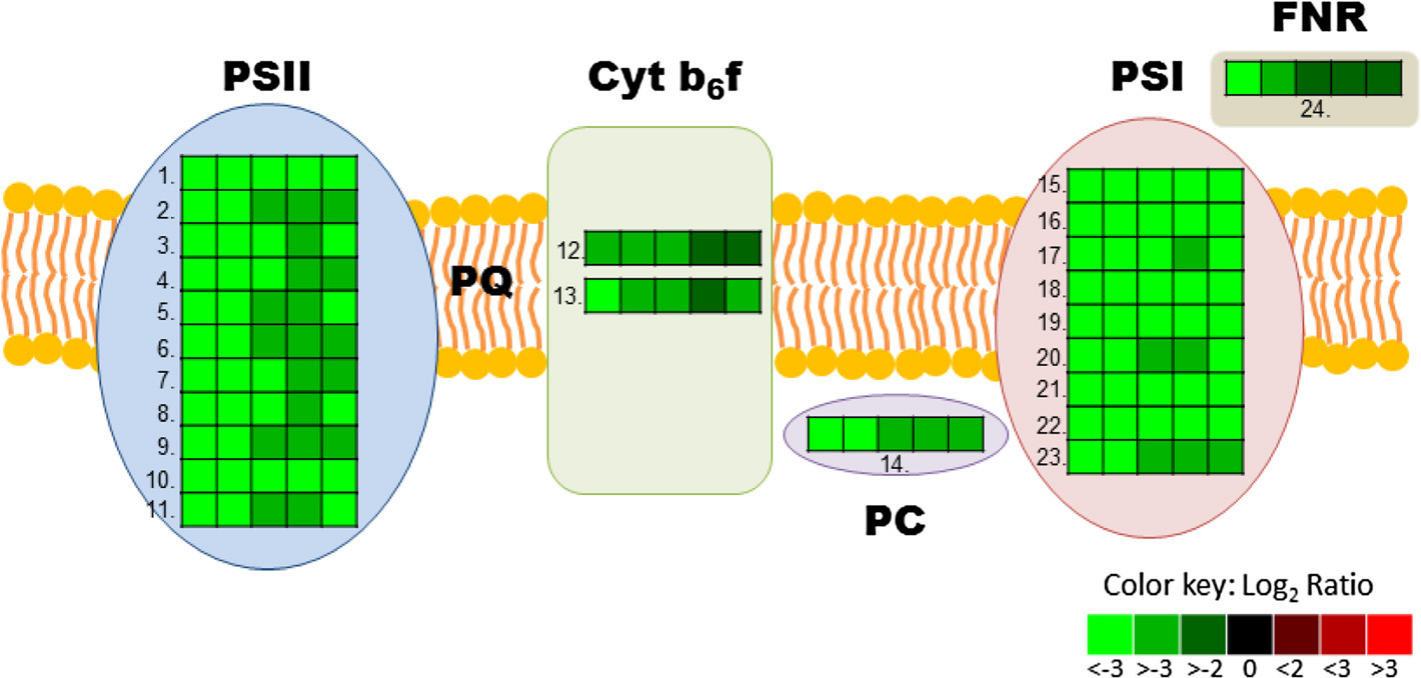
Heat map of differential gene expression involved in photosynthesis. Values given are log_2_ of the ratios between RPKM of leaves expressing *WRI1* and transformed control five days after infiltration. The order of leaf samples expressing the five different *WRI1* homologs are given for the box numbered 1: *AtWRI1*, *StWRI1em*, *PtWRI1ca*, *AsWRI1es*, and *CeWRI1tp*. 1. Light harvesting complex photosystem II subunit 6, 2. Photosystem II subunit P, 3. PsbQ subunit of the oxygen evolving complex of photosystem II, 4. Photosystem II subunit PsbX, 5. Photosystem II type I chlorophyll a/b-binding protein, 6. Photosystem II encoding the light-harvesting chlorophyll a/b binding protein CP26 of the antenna system of the photosynthetic apparatus, 7. Photosystem II 5 kD protein, 8. Involved in the light harvesting complex of photosystem II, 9. Photosystem II BY, 10. Subunit of light-harvesting complex II, 11. Photosystem II type I chlorophyll a/b-binding protein, 12. Rieske FeS center of cytochrome b6f complex, 13. Cytochrome b6f complex subunit, 14. Plastocyanin, 15. Photosystem I subunit E2, 16. Photosystem I subunit F, 17. Subunit G of photosystem I, 18. Photosystem I, subunit H2, 19. Photosystem I subunit K, 20. Protein predicted by sequence similarity with spinach PsaD to be photosystem I reaction center subunit II, 21. Photosystem I reaction center, sub PSI-N, 22. Photosystem I subunit O, 23. P subunit of Photosystem I, 24. Leaf-type ferredoxin:NADP(H) oxidoreductase

An important finding in this study was that the expression of *WRI1* homologs in leaves induced a severe down-regulation of transcripts encoding functions in the photosynthetic apparatus (Additional file 6), both in the light-dependent reactions (subunits and support proteins of photosystem I and II, cytochrome b6f complex, plastocyanin, a leaf-type ferredoxin:NADP(H) oxidoreductase, subunits of ATP generation complex, 5 to 66-fold changes, see Fig. 6) and the carbon fixation reactions of all three phases of the Calvin-Benson cycle (phosphoribulokinase, Rubisco, and the plastidic forms of phosphoglycerate kinase, glyceraldehyde-3-phosphate dehydrogenase, triose phosphate isomerase, Frc-bisphosphate aldolase, Frc-1,6-bis-phosphatase and ribose-5-phosphate isomerase, 3 to 18-fold changes, see Fig. 5). Thus the whole Calvin-Benson cycle was down-regulated together with the light harvesting and probably ATP generation of the photosynthetic apparatus (ATP synthase down-regulated 8-fold). In agreement with this, the transcript encoding the plastid localized triosephosphate/phosphate translocator, with a substrate specificity allowing organelle exchange of dihydroxyacetone-P, glyceraldehyde-3P, and 3-phosphoglycerate [45], was also down-regulated (3-fold) in *WRI1* expressing tissue which could indicate a decreased flux of the daily photosynthetically fixed carbon out from the plastid for further sucrose synthesis [46].

One example where functional duality of enzymatic activities became obvious was for Frc-bisphosphate aldolase with one up-regulated and one down-regulated transcript (Fig. 5). Since this enzyme catalyzes the reversible conversion of both Frc-bisphosphate (found in both glycolysis and Calvin cycle) and sedoheptulose-bisphosphate (only in the Calvin cycle) into dihydroxyacetonephosphate, it can be speculated that the up-and down-regulated transcripts (closest homologs, At2g01140 and At4g38970, respectively) actually represent genes encoding aldolase with importance for glycolysis and the Calvin cycle, respectively.

Transcripts encoding enzymes in starch synthesis (plastidic phosphoglucomutase, the large subunit of ADP-Glc pyrophosphorylase, and starch synthase 4) were down-regulated (3 to 7-fold) while that of β-amylase, important for starch degradation, was instead up-regulated 6-fold (Fig. 5). Increased gene expression and enzyme activity of β-amylase was previously observed during development of oil-seeds of Arabidopsis and rape indicating the importance of starch degradation to yield carbon precursors for oil accumulation in these species [7, 47]. Starch breakdown and increased glycolysis was also correlated to increased FA synthesis at transcript level in the cambium of aspen upon developmental transition to dormancy [25].

The expression of the different *WRI1* homologs in leaves induced down-regulation of many genes involved in chloroplast FA desaturation and very long chain FA (VLCFA) and cutin biosynthesis (Additional file 6). Examples were transcripts encoding FAD5 (responsible for 16:0 desaturation on galactolipids and sulpholipids in chloroplasts), FAD7 (responsible for the desaturation, also in the chloroplast, to 16:3 and 18:3 FAs found on galactolipids, sulpholipids and phosphatidylglycerol), LACS1 (CER8), LACS2 long-chain acyl-CoA synthetases related [48], CER6 (which has a major role in the production of 26 and 28 carbon FAs [49]), and a CER26-like HXXXD acyltransferase (which could have a role in elongation of VLCFA longer than 30 carbons [50]). In addition, genes annotated as coding for modifier functions of cutin were also down-regulated like CER1 which is suggested to be an aldehyde decarbonylase [51], and DCR (defective cuticle ridges), which is a HXXXD acyl-transferase [52]. Furthermore, a gene homologous to glycerol-3-phosphate acyltransferase shown to be critical for acyl transfer in cutin biosynthesis [53] was down-regulated. Possibly genes encoding P450 proteins corresponding to functions of importance for cuticle development as CYP86A2 [54] were also among down-regulated genes in leaves expressing *WRI1* homologs. Among all those, the transcript encoding FAD5 was the most severe down-regulated (53-fold), with other transcripts showing 4 to 7-fold changes. Interesting and in agreement with our results, a reduction of gene expression encoding one of the chloroplast desaturases, FAD7, was previously observed in leaf tissue expressing *AtWRI1* [31]. In the same publication it was noted that in the presence of increased TAG synthesis there was a severe reduction in surface lipids. Our results suggests that the reduction in surface lipids they observed would not be due to a competition for substrates but rather a function of reduced gene expression of transcripts related to surface lipid synthesis in response to expression of *WRI1.*

All these changes induced by *WRI1* expression imply that many of the transcripts encoding enzymes giving typical characteristics of a source tissue (photosynthesis, starch synthesis, and leaf surface lipid synthesis) were highly down-regulated, suggesting a shift into a heterotrophic tissue with low photosynthetic contribution. This can probably explain the observed chlorosis of leaves expressing *WRI1*. It can be speculated that WRI1 mediates repression of photosynthetic source functions also during seed development.

#### Carbons from oil recycled to plastid

In oil-accumulating sink tissue such as a seed, the sucrose from photosynthesizing leaves will feed the developing seed allowing for a continuous carbon flow into storage product accumulation. However, when expressing a transcription factor that, directly or indirectly, not only induce oil synthesis but also reduce photosynthesis, the sucrose available for the increased FA synthesis is likely to become limiting. In fact, transcripts encoding an α/β hydrolase with TAG lipase activity (yielding glycerol and free FAs) was highly up-regulated (192-fold) while glycerol kinase (thought to play a role for the catabolism of glycerol during post-germinative growth and leaf senescence [55]), and several enzymes involved in β-oxidation of FAs (acyl-CoA oxidase, multifunctional protein, and 3-ketoacyl-CoA thiolase) were also up-regulated 3 to 6-fold (Additional file 6). Furthermore, a transcript encoding malate synthase, involved in the closely connected glyoxylate cycle that produces malate from degraded FAs, was also highly up-regulated (78-fold). To be available for FA synthesis, malate can then either be translocated into the plastid in exchange for phosphate [56, 57] where it can be converted to pyruvate by malic enzyme, or cytosolic conversion of malate by malate dehydrogenase and phosphoenolpyruvate carboxykinase yields phosphoenolpyruvate which can be transported into the plastid by the phosphoenolpyruvate/phosphate translocator. Several transcripts encoding enzyme activities in both these routes (phosphoenolpyruvate carboxykinase, phosphoenolpyruvate/phosphate translocator, and malic enzyme, Fig. 5) were up-regulated (3 to 9-fold) in *WRI1* expressing leaf tissue as compared to transformed control. Our results therefore suggest that the new sink-like tissue induced in the leaf by expression of *WRI1* is experiencing carbon source limitations. In the absence of sucrose import this probably triggers the up-regulation of transcripts encoding enzymes in both TAG and FA degradation in the leaf in attempt to yield carbons that can be recycled back, either as malate or phosphoenolpyruvate, into glycolysis. An alternative or additional explanation could be that increased amount of TAG in a leaf mesophyll cell, without proper packaging with oil-body proteins, is more prone to mobilization which induces up-regulation of transcripts encoding enzymes of TAG degradation. This will in turn yield free FAs that can be toxic to the cell if accumulating [58], which can explain the up-regulation of transcripts encoding functions in FA degradation. (In connection to this, see discussion below regarding the time-study of free FA levels in leaves).

As mentioned earlier, the highest total oil content of leaves achieved in our study (2.2 % by dw, Fig. 2) was relatively low as compared to in other studies where the stable expression of combination of the transcription factor *WRI1* with other genes involved in TAG assembly and storage (the ‘push-pull-protect’ approach) showed that much higher oil content is possible to achieve (15 % by dw) in *N. tabacum* leaves [31]. Interestingly, the promoter chosen for *AtWRI1* expression in that study was that of Arabidopsis rubisco, with the rational of achieving a diurnal expression pattern. However, the down-regulation of photosynthesis and the Calvin-Benson cycle induced upon *WRI1* expression in this study suggests that WRI1 itself might be attenuating the expression of WRI1 from the rubisco promoter. It is possible that this plausible self-regulating effect of the *AtWRI1* expression could be another key to the high levels of TAG accumulated without apparent negative effects on plant vigor in that study.

#### Additional differentially expressed genes of potential importance for seed development

A number of genes were differentially expressed in leaves upon *WRI1* expression where the closest homologs in Arabidopsis are expressed during seed development or corresponding proteins are of importance for proper seed development. For example, transcripts annotated as encoding trehalose-6-phosphate synthase were up-regulated 30-fold (Additional file 6). Trehalose-6-phosphate is thought to be a signal of sugar status in plant storage tissues and the synthase is of crucial importance for embryo development in Arabidopsis [59–61]. Transcripts encoding proteins of completely unknown function but where the closest Arabidopsis homolog is expressed during seed development were found, exemplified by a transcript annotated as closest homolog to At2g22660 (DUF1399) (Additional file 8) which was highly up-regulated (16-fold) in *WRI1* expressing tissue (Additional file 6).

For another transcript which was also highly up-regulated (215-fold), the closest homolog in Arabidopsis was At1g43850 encoding SEUSS, a transcriptional adaptor of importance for embryonic development [62]. There were additional transcripts annotated as nucleic acid binding or transcription factors although their functions are unknown and gene families so large that closest homologs in Arabidopsis were difficult to discern. Only one transcript related to genes encoding oil body proteins was up-regulated by the *WRI1* homologs which closest homolog was At1g178101 encoding a putative aquaporin [63].

### Time study of leaves expressing *AtWRI1*

**Fig. 7:**
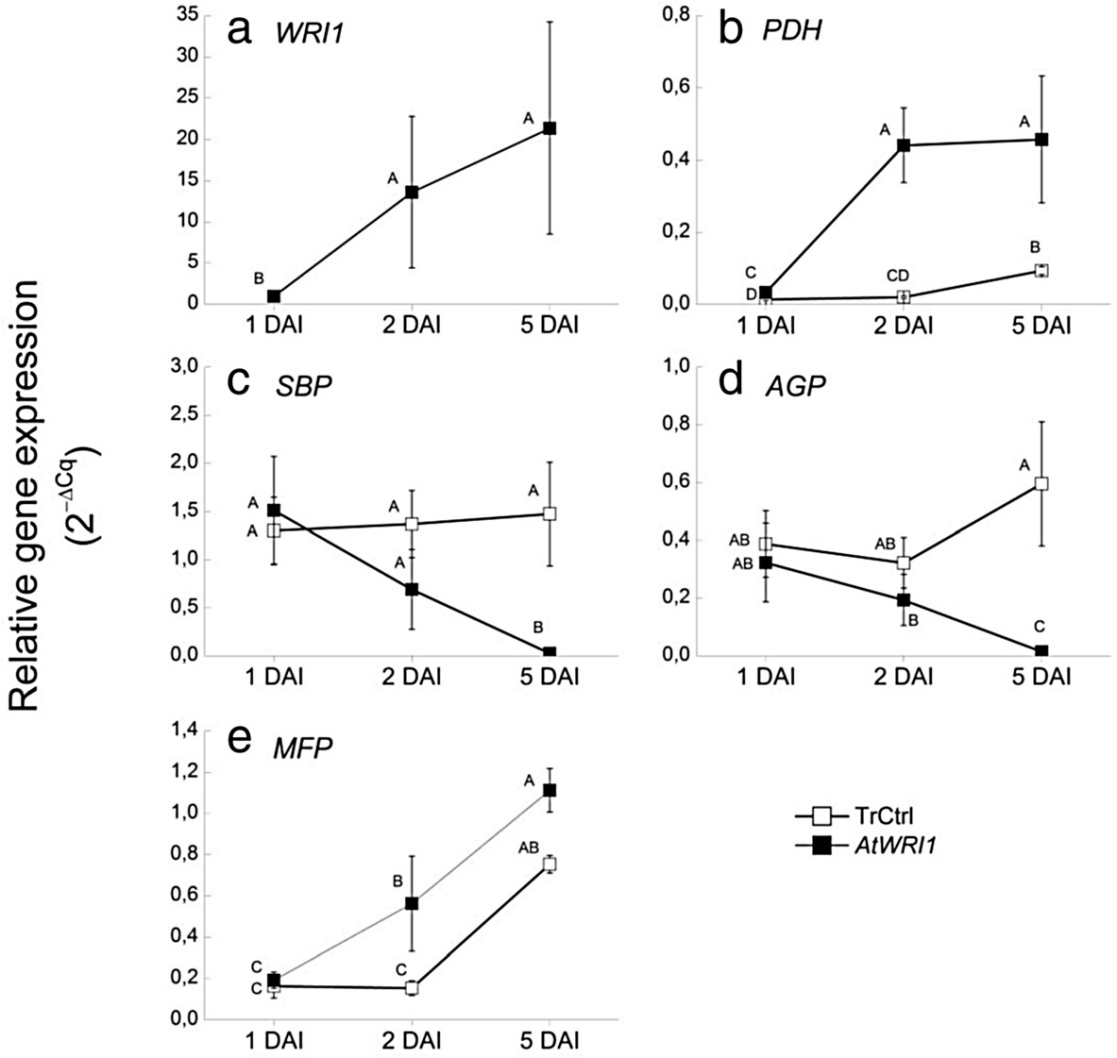
RT-qPCR time study of leaves expressing *AtWRI1*. Expression levels of *AtWRI1* (**a**) and a subset of genes encoding enzymes selected as ‘markers’ for core metabolic pathways: PDH; pyruvate dehydrogenase for glycolysis (**b)**, SBP; sedoheptulose-bisphosphatase for Calvin cycle (**c**), AGP; ADP-Glc pyrophosphorylase for starch synthesis (**d**), and MFP; multifunctional protein for fatty acid degradation (**e**) from one to five days after infiltration (DAI). Gene expression levels (2^-ΔCq^) were determined relative to the reference gene L23. TrCtrl; transformed control. Results are the mean from three biological replicates ± standard deviation. Letters distinguish significant different means according to Tukey’s test at level *P* ≤ 0.05

Some of the transcriptional transitions observed in leaves induced, directly or indirectly, by the transient expression of all the different WRI1 homologs were unexpected, such as the effect on transcripts encoding photosynthesis, starch metabolism, and fatty acid degradation. Therefore, the effects of the transient expression of *AtWRI1* in *N. benthamiana* leaves were physiologically characterized in more detail in a time study from one to five days after infiltration (DAI). Non-transformed control leaves were also analyzed in this time-study to confirm it was not physiologically different to transformed control (with *p19* and *GFP* constructs only). The expression levels of *AtWRI1* and a subset of genes encoding enzymes selected as ‘markers’ for core metabolic pathways (glycolysis, Calvin cycle, starch synthesis, and fatty acid degradation) were also determined using RT-qPCR (Fig. 7). Gene expression levels were determined relative to the gene L23 that was recently suggested to be a suitable reference in *N. benthamiana* leaves [64] and which also from our transcriptome data set was confirmed to be stable between treatments and with an expression level in a suitable range in comparison to target genes.

#### Increased *AtWRI1* expression and TAG content from two DAI

**Fig. 8:**
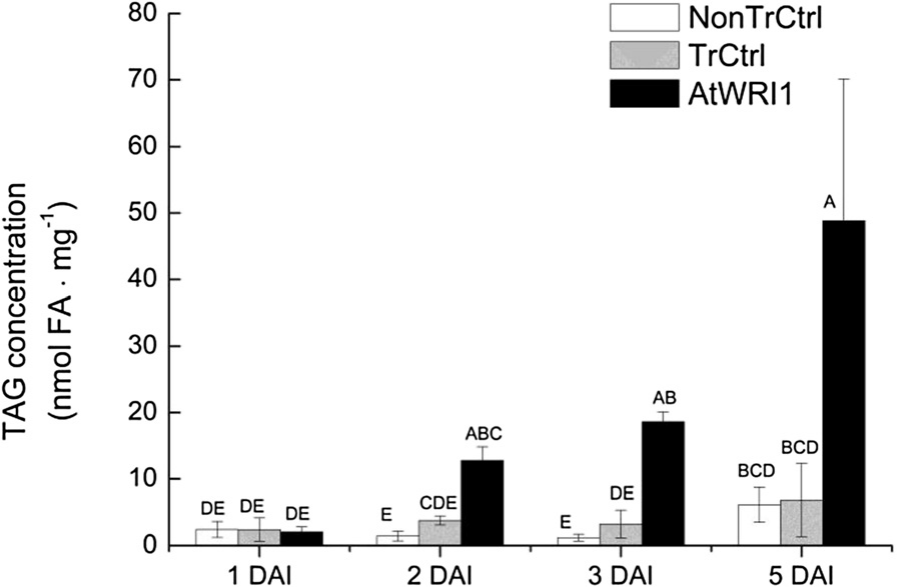
Time study of triacylglycerol concentrations in leaves expressing *AtWRI1*. Given as nmol fatty acids (FA) per mg dry weight (dw) from one to five days after infiltration (DAI). TrCtrl; transformed control, NonTrCtrl; non-transformed control. Results are the mean from three biological replicates ± standard deviation. Letters distinguish significant different means according to Tukey’s test at level *P* ≤ 0.05

*AtWRI1* expression was barely detectable in leaves at one DAI but was much increased already from two DAI as compared to in transformed control and non-transformed control, and was only slightly further increased (but not statistically significant) at five DAI (Fig. 7a). Triacylglycerol content of leaves expressing *AtWRI1* was significantly increased already by two DAI, even though it continued to increase up to five DAI (Fig. 8). This gives insight to how fast the metabolic effect generated by a constitutively expressed transcription factor in leaves can actually be observed. The expression level of the gene encoding pyruvate dehydrogenase (used as a marker for glycolysis) showed a pattern very similar to that of *AtWRI1* (Fig. 7b) which is well in accordance with that this gene is considered to be a direct transcriptional target of AtWRI1 [10].

#### Decreased photosynthetic capacity of leaves

**Fig. 9:**
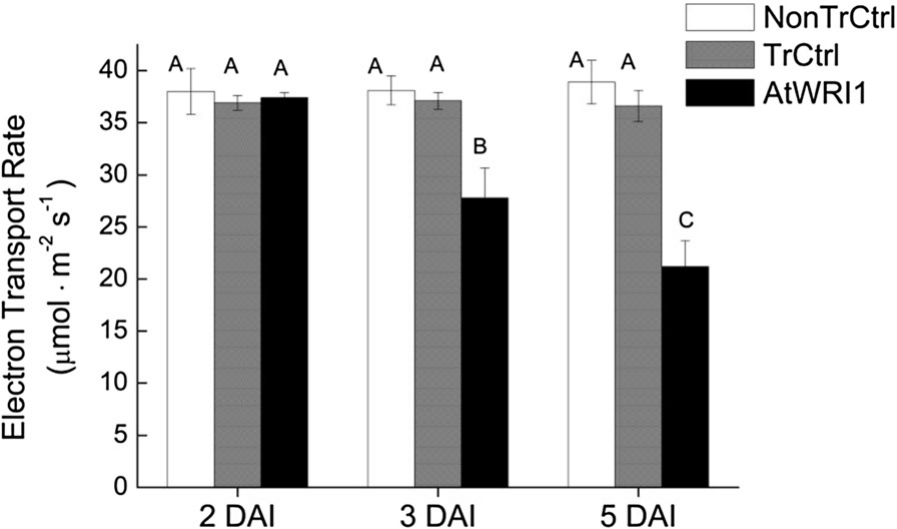
Time study of photosynthetic capacity of leaves expressing *AtWRI1*. Electron transport rate from chlorophyll fluorescence measurement in leaves two to five days after infiltration (DAI). TrCtrl; transformed control, NonTrCtrl; non-transformed control. Results are the mean from three biological replicates ± standard deviation. Letters distinguish significant different means according to Tukey’s test at level *P* ≤ 0.05

Fluorescence efficiency measurements clearly showed decreased photosynthetic capacity in leaves expressing *AtWRI1* as compared to both non-transformed leaves and transformed control from three DAI (Fig. 9) which was well in agreement with the observed severe down-regulation of many transcripts encoding different parts of the light-dependent reactions of photosynthesis (see above) as well as down-regulation of the gene used as a marker for the Calvin cycle (sedoheptulose-bisphosphatase, Fig. 7c). This result also verified that transformed control (leaves infiltrated with only *p19* and *GFP* constructs, see methods) was physiologically indistinguishable from non-transformed control (Fig. 9). Starch content was measured over time (Additional file 9) but no significant decrease could be detected. This implies that the previously described transcriptional changes observed at five DAI that were involved in photosynthesis reactions (light harvest reactions and Calvin cycle) and starch metabolism (decreased synthesis and increased degradation) were not detectable at the metabolic level of starch, at least not over the time period studied. In agreement with this, the level of the transcript encoding ADP-Glc pyrophosphorylase (catalyzing the first committed step in starch synthesis) was only statistically distinguishable from transformed control as late as by five DAI (Fig. 7d). The effect on starch levels in leaves expressing WRI1 could therefore probably not be expected until after the time period studied (i.e. after five DAI).

#### No increase in levels of free FAs in leaves

The observed up-regulation of transcripts encoding enzymes involved in TAG and FA degradation could, as discussed above, have several different explanations. It was of interest to know if leaves expressing *WRI1* had increased levels of free FAs which can have negative physiological effects [58]. This could be a reason to the observed up-regulation of transcripts encoding functions in FA degradation. From total lipid analysis in our study it was recognized that all lipids except the polar fraction were increased upon expression of *WRI1* homologs in leaves five DAI (Fig. 2). The increase of lipids in the ‘Rest’ fraction could therefore potentially include increases of free FAs, even though not visually detectable on TLC plates (Additional file 3). However, analysis of free FAs in leaves expressing *AtWRI1* showed no significant increase between one and five DAI (Additional file 10), implying that free FA levels were either not increased, or degraded in pace with the release of free FAs thereby keeping levels low. The gene expression level of multifunctional protein, used as a marker for β-oxidation of FAs, was significantly increased in leaves expressing *AtWRI1* already from two DAI and onwards, as compared to transformed control (Fig. 7e) which suggests a relatively fast response to the WRI1 mediated changes in the leaf.

## Conclusions

This study gives for the first time an overview on the transcriptional transitions in leaf tissue upon expression of the transcription factor WRI1. Expression of *WRI1* homologs from five diverse species and tissues in leaf all induced oil accumulation and the same general shifts in gene expression programs with down-regulation of transcripts encoding typical source tissue functions and up-regulation of those encoding typical sink tissue functions. The severe down-regulation observed for transcripts encoding photosynthetic functions was confirmed physiologically with a significant decreased photosynthetic capacity observed of leaves expressing *AtWRI1*. Altogether this study revealed a wider range of effects caused, directly or indirectly, by WRI1 than previously suggested. These changes are reminiscent of the transcriptional and metabolic transitions detected between the vegetative phases of seed development and maturation in Arabidopsis; from high photosynthetic contribution, starch accumulation, and cytosolic metabolism of phosphoenolpyruvate, to oil biosynthesis with decreased photosynthesis and plastid uptake and metabolism of phosphoenolpyruvate, during the course of seed development [7, 15, 44, 47]. However, leaves with *WRI1* expression did not only accumulate oil but transcriptional changes indicated that functions encoding TAG and FA degradation was up-regulated, probably in attempt to feed the increased need for carbons into FA and oil synthesis thus creating a futile cycle. This could explain why high oil content in leaf tissue cannot be achieved by high and constitutive transcriptional activation by WRI1. Most likely this response is due to an incomplete shift from source to sink tissue and a lack of induction of sucrose import.

It is intriguing that expression of *WRI1* alone can induce these large transcriptional and physiological changes in a leaf, and future studies need to functionally dissect the different roles of WRI1 and upstream transcriptional actors previously suggested to take part in seed development in general [65]. Moreover, identification of the regulatory networks present in other types of oil-storing plant tissues in which WRI1 is known to act in will be of utmost importance for the development of high-yielding oil crops in the future.

## Methods

### Plant material

All plants were grown in controlled growth chambers (Biotron, SLU-Alnarp, Sweden) under fluorescent light (200 μmol∙m^−2^∙s^−1^) and with the following conditions: *N. benthamiana*; 15.5/8.5 h light/dark photoperiod with 25/20 °C at 60 % humidity. Oat (*Avena sativa* L., cv. Matilda, Lantmännen, Svalöv, Sweden) with 10 % oil in grains; 16/8 h light/dark, at 21/18 °C at 60 % humidity. Yellow nutsedge (*Cyperus esculentus* L. var. *sativus* Boeck) was grown in an aeroponic system as previously described [18].

### Cloning of genes

*WRI1* homologs from oat and nutsedge were cloned from grain endosperm and tuber parenchyma, respectively. Oat endosperm was squeezed out from grains (from which the embryo and scutellum had first been removed to minimize tissue contamination) at mid-stage of development during active endosperm oil deposition (approximately 14 days post anthesis). Nutsedge tubers were harvested 15 days after the onset of tuber initiation during active oil deposition [18]. Total RNA was extracted from tissues using Plant RNA Reagent (Invitrogen, Carlsbad, USA) from which cDNA was synthesized and amplified using available kits either with a 5′-3′-RACE approach (for oat, Clontech, Mountain View, USA) or a standard procedure using modified primers to later be used for 454 sequencing (for nutsedge, Invitrogen, Carlsbad, USA). Gene specific primers were designed based on contig transcript sequences annotated to represent homologs to *AtWRI1* from previous 454-sequencing [unpublished results, 22]. The *AtWRI1* from embryo as well as the two homologs from potato and poplar were synthetically ordered (Eurofins, Ebersberg, Germany) based on available genomic resources. Full-length *WRI1* homologs were individually inserted to binary vectors (pART27, pK2GW7, or pXZP393) behind the 35S promotor and transformed into *Agrobacterium tumefaciens* (strain GV3101). The gene constructs with *p19* viral suppressor protein to improve gene expression and *GFP* for positive control and visual localization of gene expression were kindly provided by Dr C. Wood [35]. Full length cDNA sequences of the respective *WRINKLED* homologs were subjected to CLUSTAL 2.1 Multiple Sequence Alignment with default settings and circular cladogram created by Tree construction using Neighbor Joining and Bootstrapping with 1000 replicates on CLC Main Workbench 7.1.6 (CLC bio, Aarhus, Denmark).

### Agroinfiltration of tobacco leaves

Agroinfiltration of leaves (six weeks old, 6 h into the light period) was done as previously described by Wood et al. [35]. The *WRI1* homologs were individually combined with *p19* and *GFP* cultures, the transformed control only contained *p19* and *GFP*. Only one leaf per plant was used and identified to represent the same developmental stage (approximately 7 cm in diameter), and three biological replicates were used per construct combination. All chemicals were from Duchefa (Haarlem, The Netherlands) or Sigma-Aldrich (St Louis, USA). Culture mixtures of approximately 3 mL were infiltrated into the leaf, and plants were then incubated for five days in growth chambers. Only the leaf area visually identified to show GFP expression under UV-light was used for sampling (6 h into the light period) and was split into two; one for RNA extraction (~150 mg FW) and the other for lipid analyses (~900 mg FW). For the time-study of leaves expressing *AtWRI1*, the leaf area with GFP expression was instead divided into three; one for starch analysis, one for RNA extraction, and one for lipid analysis. Samples were snap frozen in liquid nitrogen and stored in −80 °C until further analyses. Prior to sampling, photosynthetic capacity of leaves was measured as electron transport rate using a Mini-PAM chlorophyll fluorometer (Walz, Effeltrich, Germany).

### Lipid analyses

Leaf samples were freeze dried and dry weight determined. Total lipids were extracted [66] and aliquots corresponding to 10 mg dw were separated using thin layer chromatography and quantified using gas chromatography with methyl-heptadecanoate as internal standard as previously described [67].

### Starch analyses

Dried plant material (80 °C for 48 h) was ground with mortle and pestle to a fine powder. Starch concentration was determined enzymatically on approximately 20 mg leaf dw per biological replicate using the Total starch determination kit (K-TSTA 07/11, Megazyme, Wicklow, Ireland) according to recommended procedure except that samples were first washed with 80 % ethanol at 80 °C to remove soluble sugars.

### Transmission electron microscopy

Leaves of *N. benthamiana* expressing the oat and Arabidopsis *WRI1* homologs where chosen for transmission electron microscopy (TEM). Leaf samples were taken five days after infiltration, 6 h into the light period. One leaf sample was used for both TEM and lipid analyses to determine TAG content as described above (approximately 1 % of DW for both *AtWRI1* and *AsWRI1* leaf samples). Small pieces were cut from the leaf under fixation medium (2 % paraformaldehyde (w/v), 2.5 % glutaraldehyde (v/v) in 0.1 M Sorensen’s sodiumphosphate buffer pH 7.2) to minimize air contact. Fixative was infiltrated into leaf pieces by repeated vacuum pumping in a desiccator. Further processing for TEM was performed as described previously [68].

### RNA extraction and sequencing

Total RNA from frozen leaf material was extracted by homogenizing material in Plant RNA Reagent according to instructions (Invitrogen, Carlsbad, USA) using a plastic pestle. RNA integrity and concentration was measured using Experion RNA StdSens analysis kit (BioRad, Hercules, USA) before pooling proportional RNA amounts from two biological replicates. Total RNA was DNAse treated (Turbo DNase, Ambion, Carlsbad, USA), diluted to a final concentration of 500–1000 ng∙μL^−1^, and sent for Illumina sequencing (BGI, Shenzhen, China). Preparation of libraries was performed at BGI according to their routines and sequencing was done on the Illumina HiSeq™2000.

### Bioinformatics and data analyses

Quality passed reads from the respective libraries were mapped to downloaded Niben genome v0.4.4 transcripts (ftp://ftp.solgenomics.net/genomes/Nicotiana_benthamiana/annotation/Niben044). Total number of reads ranged from 22,801,552 to 24,386,511 for leaves expressing *PtWRI1ca* and *CeWRI1tp*, respectively, with leaves expressing the other *WRI1* homologs having number of reads in between those. Proportions of mapped sequenced reads to the *N. benthamiana* reference genome ranged from at lowest 63.8 % to highest 79.6 % for leaves with *AsWRI1es* and *StWRI1em*, respectively. Pairwise comparison (ratio) of RPKM (reads per kilobases and million mapped reads) values of each transcript in *WRI1* homolog expressing leaf tissue to that in transformed control yielded an initial file containing transcripts where FDR ≤ 0.001 and a log_2_ ratio ≥1). Differential gene expression is given as log_2_ of ratios of RPKM in leaves expressing the *WRI1* homologs as compared to transformed control (log_2_ ratio of +3 means that a transcript is 2^3^ = 8 times higher expressed). Transcripts showing a differential expression as compared to transformed control with a *P*-value close to zero (<10^−307^) were extracted to an individual table (Additional file 6 with log_2_ ratios in bold font) in which also differential expression values for all other leaf samples were included (even though having a *P*-value above this threshold, log_2_ ratios in italic font in Additional file 6). For all genes discussed in this study, the annotation made by BGI was complemented with the closest homologs of *N. benthamiana* transcipts to Arabidopsis genes [69].

For determination of *WRI1* expression, respective *WRI1* sequences used for infiltration were introduced among Niben genome v0.4.4 transcripts in a new reference file. Illumina sequence reads representing transcripts for each of the leaf samples with expressed *WRI1* homologs were subsequently mapped to the new reference file using CLC Genomics Workbench 7.0.4 (CLC bio, Aarhus, Denmark) using the RNA-Seq analysis module at default settings.

### RT-qPCR

Frozen leaf samples taken from the time-study on leaves expressing *AtWRI1* (three biological replicates) were ground to a fine powder in 1.5 mL plastic tubes using metal beads in a mixer mill (MM400, Retsch, Haan, Germany) with care taken to not let material thaw at any time. Total RNA was extracted and 10 μg was DNase treated (using the same kits as described above). For cDNA synthesis, 1.2 μg DNase treated total RNA was reverse-transcribed using Superscript III First-Strand Synthesis Supermix for RT-qPCR (Invitrogen, Life Technologies, Carlsbad, CA, USA). Transcript quantification was made on a BIO-RAD C1000 Thermal Cycler, CFX 96 Real-Time System (California, USA) using Maxima SYBR Green/ROX qPCR Master Mix (2X) (Thermoscientific, Life Technologies, Carlsbad, CA, USA), 2.5 μL of 1:10 diluted cDNA and 0.3 μM primers in a final reaction volume of 25 μL run in white PCR tubes (Starlabs, SaveenWerner, Limhamn, Sweden) using a program with initial 95 °C for 10 min followed by 40 cycles of 95 °C for 15 s, 60 °C for 30 s, 72 °C for 30 s. Primers used are listed in Additional file 11 where forward primers were designed to span the exon borders (for all except *L23* and *AtWRI1* for which it was not technically possible or meaningful, respectively) to minimize amplification of genomic DNA. All used primers gave single PCR product as determined by visual inspection of agarose gel electrophoresis. C_q_ values were calculated using automatic single threshold in the Bio-Rad CFX Manager and values between 18–30 cycles were regarded an appropriate quantification window. Melting curves for PCR products were inspected to confirm identical melting temperature for each specific product. Expression levels of transcripts were calculated relative to *L23* [64] according to the equation Relative Expression = 2^-ΔCq^ where ΔC_q_ = C_q_(target) - C_q_(reference). Mean values of three technical replicates where used for each biological replicate.

### Statistical analyses of data

Treatment effects on data obtained from chemical analyses and RT-qPCR were analyzed by analysis of variance (ANOVA) using the general linear model (MINITAB 16; Minitab, State College, PA, USA) in which all factors were fixed. Significant differences between treatments were calculated using pairwise comparisons with Tukey’s test at level *P* ≤ 0.05. When necessary, data was first transformed by the logarithmic transformation before the ANOVA and Tukey’s test to stabilize the variance and obtain an approximately normal distribution of the residuals required for valid statistical inference.

### Availability of supporting data

The sequences used in this study have been submitted to the Sequence Read Archive at NCBI (Accession numbers: SRX1079394 (transformed control leaves), SRX1079397 (leaves expressing *AtWRI1*), SRX1079426 (*StWRI1em*), SRX1079428 (*PtWRI1ca*), SRX1079429 (*AsWRI1es*), and SRX1079431 (*CeWRI1tp*).

## Additional files

Additional file 1:
**Complete cDNA sequences of** ***WRI1*** **homologs.** Sequences are from potato embryo (*StWRI1em*), poplar stem (*PtWRI1ca*), oat endosperm (*AsWRI1es*), and nutsedge tuber parenchyma (*CeWRI1tp*). (DOCX 15 kb) Additional file 2:
**Amino acid homology of WRI1 homologs.** Comparison of WRI homologs from Arabidopsis (AtWRI1, 3, and 4), poplar stem (PtWRI1ca), potato embryo (StWRI1em), oat endosperm (AsWRI1es), nutsedge tuber parenchyma (CeWRI1tp), and tobacco (NbWRI1) in regions spanning the two AP2/EREBP DNA-binding domains. Black means identical, shaded means similarity. Homology is measured as compared to Arabidopsis WRI1 sequence. (JPEG 236 kb) Additional file 3:
**Photo of thin-layer chromatography plate.** Lipid classes of total lipid extracts corresponding to 10 mg dw from leaves transiently expressing *WRI1* from Arabidopsis embryo (*AtWRI1*), potato embryo (*StWRI1em*), oat endosperm (*AsWRI1es*), poplar stem (*PtWRI1ca*), and nutsedge tuber parenchyma (*CeWRI1tp*) five days after infiltration. Red arrows indicate polar lipids (PL), diacylglycerol (DAG) and triacylglycerol (TAG; oil). Results are from three biological replicates. (TIFF 181 kb) Additional file 4:
**Dry matter content of leaves.** Dry matter as % dw out of fresh weights of leaves transiently expressing *WRI1* from Arabidopsis embryo (*AtWRI1*), potato embryo (*StWRI1em*), oat endosperm (*AsWRI1es*), poplar stem (*PtWRI1cm*), and nutsedge tuber parenchyma (*CeWRI1tp*) five days after infiltration. Results are the mean from three biological replicates ± standard deviation. Letters distinguish significant different means according to Tukey’s test at level P ≤ 0.05. (JPEG 2142 kb) Additional file 5:
**Number of differentially expressed genes (DEGs).** Up-regulated and down-regulated genes in leaves expressing *WRI1* from Arabidopsis embryo (*AtWRI1*), potato embryo (*StWRI1em*), oat endosperm (*AsWRI1es*), poplar stem (*PtWRI1ca*), and nutsedge tuber parenchyma (*CeWRI1tp*) in pairwise comparisons to transformed control five days after infiltration. (JPEG 4995 kb) Additional file 6:
**Log**_**2**_ **ratios and RPKM values of differentially expressed genes.** Table of genes shown to be differential regulated (according to chosen criteria, see Material and Methods) in leaves expressing *WRI1* from Arabidopsis embryo (*AtWRI1*), potato embryo (*StWRI1em*), oat endosperm (*AsWRI1es*), poplar stem (*PtWRI1cm*), and nutsedge tuber parenchyma (Ce) in pairwise comparisons to transformed control. Genes are grouped into functional categories (CAR; carbohydrates, CYT; cytochromes, DEF; defence, FAS; fatty acid synthesis, GLY; glycolysis, LIP; lipids, MRE; mitochondrial respiration, OTH; other genes, PHO; photosynthesis, PPP; pentose phosphate pathway, STA; starch, STR; stress, TRA; transport, TRF; transcription factors, UNK; unknown. At numbers represent closest Arabidopsis gene homologs of *N. benthamiana* transcripts from the TAIR database. (XLSX 329 kb) Additional file 7:
**Gene expression pattern of At2g40610 in Arabidopsis.** Expression pattern in different tissues according to output image from the developmental series of the Arabidopsis eFP browser with settings in absolute mode. (JPEG 84 kb) Additional file 8:
**Gene expression pattern of At2g22660 (DUF1399) in Arabidopsis.** Expression pattern in different tissues according to output image from the developmental series of the Arabidopsis eFP browser with settings in absolute mode. (JPEG 91 kb) Additional file 9:
**Time study of starch concentration in leaves expressing** ***AtWRI1*** **.** Concentration given as percentage on dry weight (dw) basis from one to five days after infiltration (DAI). TrCtrl, transformed control. Results are the mean from three biological replicates ± standard deviation. Letters distinguish significant different means according to Tukey’s test at level P ≤ 0.05. (JPEG 2863 kb) Additional file 10:
**Time study of free fatty acid concentration in leaves expressing** ***AtWRI1*** **.** Given as nmol fatty acid (FA) per mg dry weight from one to five days after infiltration (DAI). Results are the mean from three biological replicates ± standard deviation. Letters distinguish significant different means according to Tukey’s test at level P ≤ 0.05. (JPEG 1958 kb) Additional file 11:
**Primers used in RT-qPCR analyses.** (DOCX 14 kb)

## Abbreviations

ACP: Acyl carrier protein
FA: Fatty acid
KAS: Ketoacyl-ACP synthase
PDCT: Phosphatidylcholine diacylglycerol cholinephosphotransferase
TAG: Triacylglycerol
VLCFA: Very long chain fatty acid
WRI1: WRINKLED1
