# Supplementary material for: Transcriptional transitions in Nicotiana benthamiana leaves upon induction of oil synthesis by WRINKLED1 homologs from diverse species and tissues
Source: BMC Plant Biol. 2015 Aug 8;15:192. doi: 10.1186/s12870-015-0579-1 (PMC4528408; doi:10.1186/s12870-015-0579-1)
Supplement: Additional file 1: — Complete cDNA sequences of WRI1 homologs. Sequences are from potato embryo (StWRI1em), poplar stem (PtWRI1ca), oat endosperm (AsWRI1es), and nutsedge tuber parenchyma (CeWRI1tp). (DOCX 15 kb) [file 12870_2015_579_MOESM1_ESM.docx]

**Additional file 1. Complete cDNA sequences of *WRINKLED1* (*WRI1*) homologs**. Sequences are from potato embryo (*StWRI1em*), poplar stem cambium (*PtWRI1ca*), oat endosperm (*AsWRI1es*), and nutsedge tuber parenchyma (*CeWRI1tp*).

>*StWRI1em*

ATGAAGAAATCTCCATCTTTTTCTTGCTCCTCTTCTTCTTCATCATCTTCATGCATTGAACAAATCCATGAAGAAACAGAGAAGCTAAACCTGAAGCCGAAACCAAAGCCCAGGCTGAAACGTGCTAGCCGAGCTAAAAAAATTGTAAATGCTGATTCACCAAGTAATAATTCATCTACAGCTAGAAGAAGCTCCATTTACAGAGGCGTAACCAGGCATAGATGGACAGGAAGGTATGAAGCTCACTTATGGGACAAGAGTACTTGGAATAGCATTCAGAACAAAAAAGGAAGACAAATTTATTTGGGAGCTTATGATAGTGAAGAAGCTGCTGCTAGAACTTATGATCTTGCTGCCCTTAAGTATTGGGGACCAACAACTATACTTAATTTTCCGGTGGAAAGATATAGCCAAGAGTTTGAAGAAATGCAGAGGCTAACAAAGGAAGAGTACTTAGCTTCATTAAGGAGAATGAGCAGTGGATTTTCAAGAGGTGTTTCCAAATATCGTGGCGTAGCAAGGCACCACCATAATGGTCGATGGGAGGCGCGAATTGGACGGGTCTATGGAAATAAGTATCTCTATTTGGGAACTTACAGCACTCAAGAGGAAGCAGCTGCAGCATATGATATGGCAGCAATTGAGTACAGAGGGCCTAATGCAGTCACCAACTTCGACATCAGCAGATATGCAGACCATTTGAAGAAACTTCGCGAACCAAATCTGTTAACAAAGGAGGAAAACACAGAGTCCTCTGCTGAAGTTCAATCAAATGAGGTGATCGAACAGTATCAGCCGGTTCAACAAGAAGAAAACCAATTGGATTATCAGCTAGTGGAATTAGCTGCAGAACCAATAGTAGTACCAAAGTTGGAGTTTACGCCTGCACTTGATTCTGATGAAGTGACTCAACCAAAAGTTCTGAAGTTGGAGTTTGCTGCTGATATAATGAAGGCGAAGGACCACGAGGAGGAAGAGGACTACCCGTGGATGAACATGTACTTGGACAACACATTTGATTCGCTCCCAGTTTCCGACTTCTCTCTTGACAAACCAGCTGACTTGATGGATCTTTTCAATGACAACAGCTTTGACAACAACATTGACTTCAATTTCTATGAGCAATCAAGTGAGAATGAGTTCAACCTGAATGTATTCTCCGACAGCATGATCATCGATGGGATTGAAGCTGATAATGAGGAAGTAAGGAACAATCTGTCGATTTCACCTACCTCATCATCATCCATGTCAAGAACAACATCCATTTCCAACGACATGAAAGACGATGGAGGTTTGGCATCTTCGACGATGTAA

*>PtWRI1ca*

ATGAAGAGGTCTTCGTCTTGCTCCTCCTCCTCCTCCTCTTCACCATATTGTGTGGCCTCTGAAAGCATTCAGAAGCCAAAAGTCAAACGCATTAGAAAAAACCAAAAGAGCAATCAGGGGAAATCCCATAAGAATGCTGCTGCTGCTGCCAACAGTCCTAACTCTGGCAAAAGAAGTTCCATTTACAGAGGAGTCACCAGACATAGATGGACAGGAAGGTTTGAAGCTCATCTCTGGGATAAGAGTTCATGGAACAGCATTCAAAACAAGAAGGGAAAACAAGTTTATTTGGGTGCCTATGATAATGAGGAGGCAGCCGCACACACCTACGATCTTGCTGCCCTGAAGTACTGGGGGGCAGAGACAACCTTGAATTTTCCGATAGAAACATACACAACGGAGATCGAAGAGATGCAGAGGGTGACCAGGGAAGAGTACTTGGCATCACTTAGACGGAAAAGCAGTGGATTCTCCAGAGGAGTCTCTAAGTACCGTGGGGTGGCTAGGCATCATCACAATGGTCGATGGGAAGCCAGAATTGGACGAGTTCAAGGGAATAAATATCTCTATCTTGGAACTTATAATACGCAAGAAGAGGCAGCGGCAGCATATGACATGGCAGCAATAGAATATAGAGGAGCAAATGCTGTGACCAATTTTGATGCAGGCAATTATATAGAACGGATGAGGGAGAAAGGCATCCCTATAGACCAAATCCTCCAAGAACAACAACAACAACAACAACTTGGTAACAACTCGATTGATCCCGGCATAGAAGTAGAGGCAGGAGTTGAACAACCATCACCGCAACAACAAGAGGAACAAGAGCAAAAAGTAGCTCCGTCGTTGCAAGTTCAATGCACACAGCTAGATTCAAGCTTGGATGGCGCATCTCCCATGGTTATTACGGACACCATCGAAGAGCACGAGCAAGCATGGAGCTTTTGTATGGATTCAGGATGGAATCTCACAATGCTTGATCTTCCTTTCGAAAATTCTTGCGAGCTGCCGGACTTGTTCAATCATACAGGGTTTGAAGACAACATTGACTTGATGTTTGATGCATGTTGCTATGGAAACTAA

*>AsWRI1es*

ATGAAGAGATCCCCGCCTCCGGCCCCTCCTGCAGCTCCTCCTCCTCCTCAGCCGTCTCCTTCTTCTTCCTCGCCGGCATGCTCGCCTTCCCCGTCGTCGTCTTCGTGTCCGTCCTCCTCCGACTCCTCATCGATCGTCATTCCCCGCAAGAGAGCGCGGACGCAGAAGGCCGCGAGCGGCAAGCCCAAGGCCAAGGCGTCGGCCAAGAGGCCCAAGAAAGACGCGAGCCGGAGCAGCAAGGAGACCGACGCCAATGGCGCTGCCGCCGCCGCCGGGAAGAGGAGTTCCATCTACAGGGGAGTCACAAGGCACAGGTGGACAGGCAGATTTGAGGCACATCTCTGGGACAAGAACTGCTTCACTTCCGTCCAGAATAAGAAAAAAGGGAGGCAAGTCTATCTGGGGGCTTATGATACAGAGGACGCTGCCGCTCGAGCATACGACCTTGCAGCGCTCAAATATTGGGGTTCTGAAACAATACTCAATTTCTCAGTGGAGGACTATGCCAAAGAGATGCCGGAGATGGAGGCGGTCTCAAGGGAGGAGTACCTCGCCGCCCTCCGCCGCCGGAGCAGTGGCTTCTCAAGGGGTGTCTCCAAGTACCGGGGCGTCGCCAGGCACCACCACAATGGGCGGTGGGAGGCACGAATTGGGCGGGTGCTGGGGAACAAGTACCTCTACCTGGGAACCTTTGATACCCAAGAGGAAGCAGCCAAGGCCTATGATCTTGCAGCCATTGAATACAGAGGTGCCAATGCTGTAACCAATTTTGACATTAGCTGCTACCTGGACCAGCCACAGTTGCTGGCACAACTGCAGCAGGGGCCACAGGTGGTGCCAGCATTGCAAGAGGAACTTCAACATGATGTTCAACATGATCTTCAAAATGACAATGCAGTCCAAGAGCTCAATTCAGGCGAAGTGCAGATGCCTGGTGCCATGGATGAGCCGATTGCACTGGATGACAGTACCGAATGCATCAACACCCCCTTTGAGTTTGACTTCAGCGTCGAAGAGAACCTCTGGAGCCCTTGCATGGATTATGAATTGGATGCTATTTTGGGAAATAACACCAGCAACTCGGCGAATATGAATGAATGGTTCAACGATTCCACCTTCGAGAGCAACATTGGGTGCCTGTTTGAAGGATGCTCCAACATCGACGACTGCAGCAGCAGCAAGCATTGTGCAGATCTGGCAGCATTTGATTTTTTCAAGGAAGGTGACGATAATGATTTCTCAAACATGGAAATGGAAATAACTCCTCAAGCAAATGATGTTTCATGCCCTCCAAATGATGTCTCATGCCCCCCAAAGATGATCACTGTGTGTAATTGA

*>CeWRI1tp*

ATGACTCCATTTCCCCCTCTTGAGAGGAAGAGGCCATGCCCAGCACCTCTCTCCCCTTCCTCCTCCACCTCCTCCACCTCCTTTGACAATGCATGTGGCAAGGAGAACAAGCCGAAGCCGAAGCGCGCCAAGAGAGCCAAGAACCCTTCCAAGGCCCTCCCTTCTCCAACTGGACCTTCTGGCAATGCAAAGAGGAGCTCCATCTACAGAGGAGTCACCAGGCATAGATGGACTGGGAGGTTTGAAGCTCATCTCTGGGATAAGAACTGCTGGAACTCTCTCCAGAGCAAGAAAGGCAAACAAGTTTATTTGGGAGCTTATGATACCGAAGAAGCCGCGGCGCGCACGTACGACCTCGCTGCACTCAAATACTGGGGGCCAGAGACCTTGCTGAACTTCCCTTTGGAGAGGTATGAGGCAGAGATCGAGGCCATGAAGGCAGTGTCAAGGGATGAGTACTTGGCATCACTGAGAAGAAGCAGCAGTGGCTTCTCCAGAGGAGTTTCCAAGTACAGAGGAGTGGCAAGGCACCATCACAATGGAAGATGGGAGGCCAGGATTGGAAGAGTGTTTGGCAATAAGTATCTCTACCTGGGAACATTCAGTACTCAAGAGGAAGCGGCACGGGCGTACGACCTCGCTGCGATAGAGTACCGGGGCGTAAACGCCGTGACAAACTTTGACATCAGGCAGTACATGGAGGACCATCCTGAGAAGTTCCGGCAAGAGCAAGGTCAGGCTGTGACATCTGATGAGACCAAGCCTGAGTCGAGCCCGGTGGAAGGGGCAGACATGGTGCCATCAGATGGAAATGCGGATGAGAATGAGAATGAGAATGTATTGAGTGAGGGAAGCACGGTTGAAGACCCGTGGAACGCTCTGACTGAGGAGAAGTTCCAGTTCGATCTGGATACAAACATCGACTGCCTGCTGCACGATGTCCCGTTCGAGAATGACATCGAGTGTGTCCTCAACGGGTCCGAGTTCGATGCGAACCAGCTCGAGGTGGTGAAAGGGGAGGAAATCATCATGAAGAACGCTTCATCCGAAGCACCGATCGTTTGCCTATGCGCAAATGCGAATAATGTGCATGCTTGTTTGTGCTGGTAG
