# Supplementary figures and images for: Transcriptional transitions in Nicotiana benthamiana leaves upon induction of oil synthesis by WRINKLED1 homologs from diverse species and tissues
Source: BMC Plant Biol. 2015 Aug 8;15:192. doi: 10.1186/s12870-015-0579-1 (PMC4528408; doi:10.1186/s12870-015-0579-1)

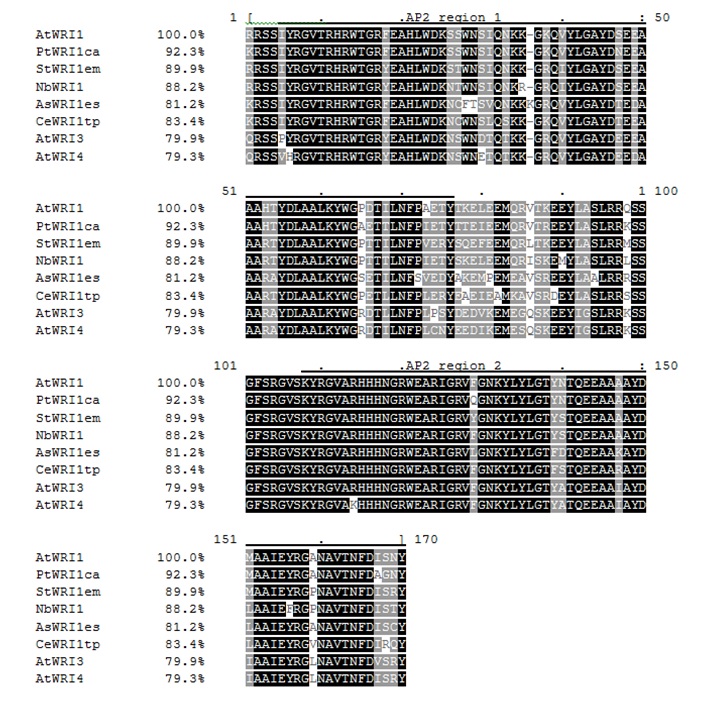

Supplement: Additional file 2: — Amino acid homology of WRI1 homologs. Comparison of WRI homologs from Arabidopsis (AtWRI1, 3, and 4), poplar stem (PtWRI1ca), potato embryo (StWRI1em), oat endosperm (AsWRI1es), nutsedge tuber parenchyma (CeWRI1tp), and tobacco (NbWRI1) in regions spanning the two AP2/EREBP DNA-binding domains. Black means identical, shaded means similarity. Homology is measured as compared to Arabidopsis WRI1 sequence. (JPEG 236 kb) [file 12870_2015_579_MOESM2_ESM.jpg]

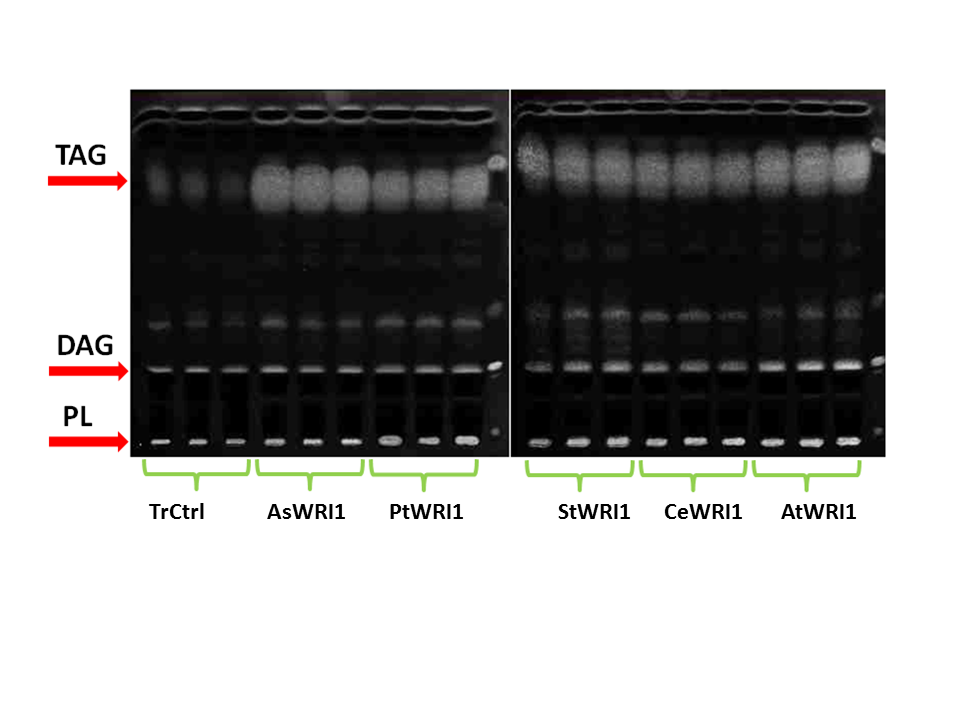

Supplement: Additional file 3: — Photo of thin-layer chromatography plate. Lipid classes of total lipid extracts corresponding to 10 mg dw from leaves transiently expressing WRI1 from Arabidopsis embryo (AtWRI1), potato embryo (StWRI1em), oat endosperm (AsWRI1es), poplar stem (PtWRI1ca), and nutsedge tuber parenchyma (CeWRI1tp) five days after infiltration. Red arrows indicate polar lipids (PL), diacylglycerol (DAG) and triacylglycerol (TAG; oil). Results are from three biological replicates. (TIFF 181 kb) [file 12870_2015_579_MOESM3_ESM.tif]

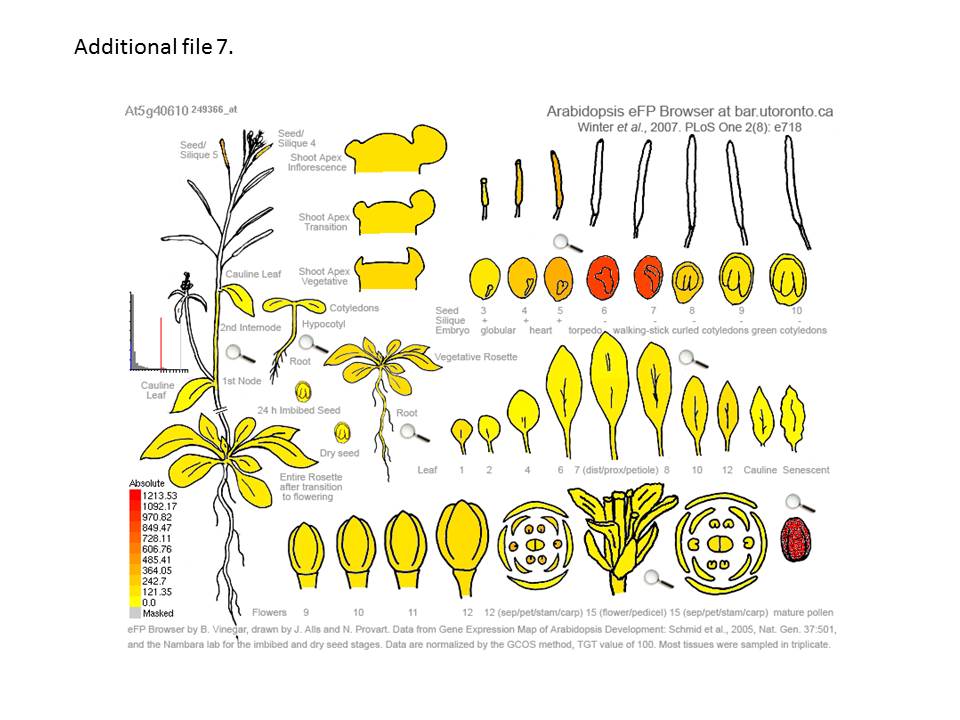

Supplement: Additional file 7: — Gene expression pattern of At2g40610 in Arabidopsis. Expression pattern in different tissues according to output image from the developmental series of the Arabidopsis eFP browser with settings in absolute mode. (JPEG 84 kb) [file 12870_2015_579_MOESM7_ESM.jpg]

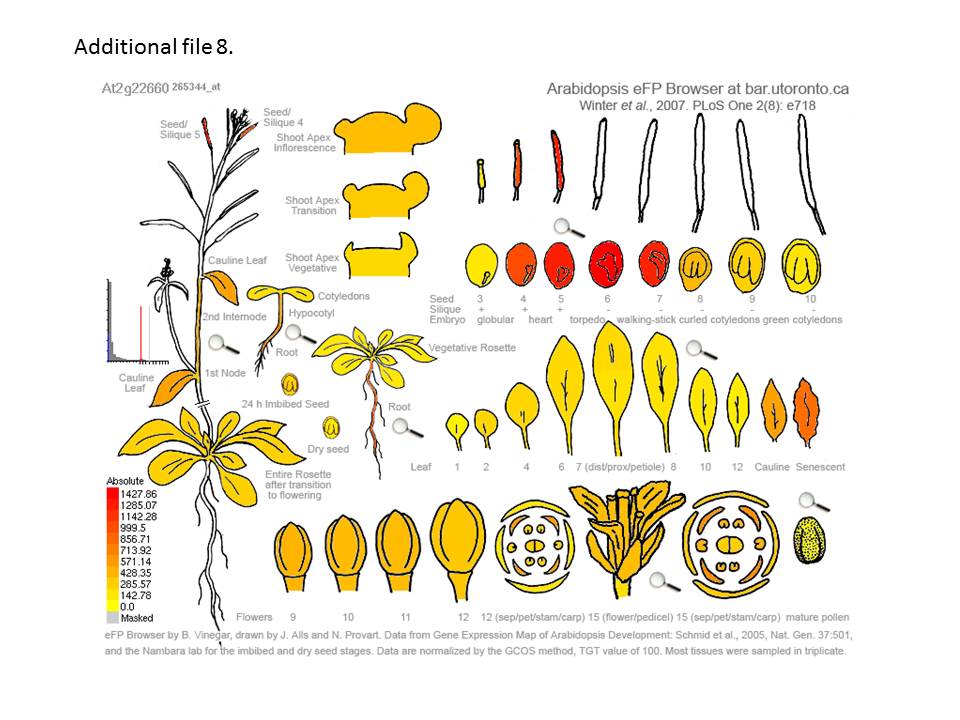

Supplement: Additional file 8: — Gene expression pattern of At2g22660 (DUF1399) in Arabidopsis. Expression pattern in different tissues according to output image from the developmental series of the Arabidopsis eFP browser with settings in absolute mode. (JPEG 91 kb) [file 12870_2015_579_MOESM8_ESM.jpg]
