# Supplementary material for: Transcriptional transitions in Nicotiana benthamiana leaves upon induction of oil synthesis by WRINKLED1 homologs from diverse species and tissues
Source: BMC Plant Biol. 2015 Aug 8;15:192. doi: 10.1186/s12870-015-0579-1 (PMC4528408; doi:10.1186/s12870-015-0579-1)
Supplement: Additional file 11: — Primers used in RT-qPCR analyses. (DOCX 14 kb) [file 12870_2015_579_MOESM11_ESM.docx]

**Additional file 11. Primers used in RT-qPCR analyses.**

| **Gene name** | **Gene transcript N. benthamiana** | **Forward 5´-3´** | **Reverse 5´-3´** | **Poduct size (bp)** |
| --- | --- | --- | --- | --- |
| Pyruvate dehydrogenase E1 beta, plastid | NbS00007894g0004.1 | TTCAGAGAGGAAAGGAAGGT | AAGTAGAAGCAGCAGCAGT | 143 |
| Multifunctional Protein (betaox) | NbS00017975g0015.1 | AAGGGTGCTCTGCTGAGTTCTA | CTTTTCCTTATCCACTCCGCT | 139 |
| Sedoheptulose-bisphosphatase, chloroplast Calvin cycle | NbS00020569g0013.1 | TCCTCCTCGATGAAGGGAAATG | GTAGTAATCGATTAGCTTGGCA | 131 |
| Large subunit of ADP-glucose pyrophosphorylase | NbS00051139g0006.1 | TTGCAGGTGAGGCTAAGTTA | CAATTGGAACAGCAGGCTTA | 141 |
| AtWRINKLED1 | - | AGACATAGATGGACTGGGAGA | ATCGTACGTATGTGCTGCTG | 135 |
| L23 (60S ribosomal protein) | NbS00019339g0015.1 | TGAGGACAACAATACCCTTG | GTCCCATCAGGCCTAATCAA | 129 |
